# Income is not an equalizer: health development inequities by ethnoracial backgrounds in California kindergartners

**DOI:** 10.1186/s12889-023-17246-7

**Published:** 2023-12-11

**Authors:** Judith L. Perrigo, E. Piper Block, Efren Aguilar, Chandler Beck, Neal Halfon

**Affiliations:** 1 Department of Social Welfare, University of California, Los Angeles (UCLA), Luskin School of Public Affairs, 337 Charles E Young Dr E, Los Angeles, CA 90095 USA; 2 Department of Pediatrics, David Geffen School of Medicine, University of California, Los Angeles (UCLA), Center for Healthier Children, Families, and Communities, Los Angeles, USA

**Keywords:** Early Childhood, Kindergarten Inequities, Early Development Instrument (EDI)

## Abstract

**Background:**

Early childhood health development is positively associated with income, but the strength of this relationship with ethnoracial background remains unclear. This study examined the extent of health development inequities among California kindergarteners based on ethnoracial backgrounds and neighborhood-level income.

**Methods:**

This cross-sectional study assessed health development inequities by analyzing neighborhood-level income, ethnoracial background, and health development data for California kindergarteners. Student-level data (*n* = 106,574) were collected through teacher report between 2010–2020 across 52 school districts and 964 schools. Student addresses were geocoded and linked to American Community Survey neighborhood income levels. Health development was measured using the Early Development Instrument, a population-level measure which includes physical health and well-being, social competence, emotional maturity, language and cognitive development, and communication skills and general knowledge domains. Outcomes included being “on-track” in each domain as well as overall health development.

**Results:**

Using a Generalized Estimation Equation with a log-link function, while accounting for interactions between ethnoracial background, income, and income-squared, we found significant health development inequities by ethnoracial background and neighborhood-level income. Regarding overall health development, as well as the physical, social and emotional domains, Black students had a lower likelihood of being on-track compared to the weighted average across income levels, whereas Asian students surpassed the weighted average. White students exhibited the steepest slope, and at the lowest income levels, their health development scores were akin to their Black and Hispanic/Latino/a low-income counterparts but resembled their Asian counterparts at higher income levels. For the general knowledge and communication domain, white students consistently had the highest likelihood of being on-track, while Hispanic/Latino/a students had the lowest likelihood across all income levels.

**Conclusion:**

This study examines health development inequities among California kindergarteners in diverse communities. Our analysis shows that the relationship between neighborhood-level income and kindergartners’ health development varies by domain and is weaker for students of color. Given the scarcity of population-level data on health development outcomes, these analyses offer valuable insights for identifying ecosystems necessitating support in promoting equitable early childhood health development.

**Supplementary Information:**

The online version contains supplementary material available at 10.1186/s12889-023-17246-7.

## Background

Lifelong health inequities originate early in life [1–3], and evidence shows that investments during sensitive early childhood developmental periods can have substantial, long-term impacts on later life [1, 4–6]. To address health inequities before they grow into debilitating patterns, it is strategically wise to consider which investments are likely to effectively and efficiently address health inequities from the start.

Without intervention, children from minoritized ethnoracial backgrounds and children from low-income neighborhoods are more likely to experience social adversities and inequities with impacts across a range of health development outcomes [7]. For instance, compared to high-income or white^1^children, low-income, Hispanic/Latino/a, and Black children tend to have poorer physical health and wellbeing [8], experience more community violence [9], and encounter greater educational disparities [10]. Research examining the link between neighborhood poverty and school readiness has consistently discovered significant achievement gaps based on this factor [11–13]. These studies underscore the potential usefulness of neighborhood poverty as one indicator to identify children requiring additional support [13]. On the other hand, when young children have equitable, high-quality interactions in early childhood (e.g., childcare centers, preschools, neighborhoods, or their homes), they are more prepared to thrive in kindergarten [14–16]. These positive experiences compound; children who succeed in kindergarten are more likely to experience better health, higher rates of academic achievement, high school completion, college matriculation, employment, income, and stable marriages, as well as lower rates of crime and teenage pregnancy [10, 17–19]. Thus, success in kindergarten provides an important signal for measuring health development trajectories because it offers a seminal population-level indicator of current and potential future health disparities. It underscores the importance of early investment for promoting better health outcomes in adolescence and adulthood [20].

Unlike many other nations, the United States has not yet adopted a universal, population-level measure of kindergarten readiness and health development administered when children start school. Canada and Australia both utilize the Early Development Instrument (EDI), which comprehensively measures the health development of kindergartners, including physical, social, emotional, language, and cognitive development [21]. Despite an absence of federal investments, the EDI has been implemented in parts of the United States through local initiatives, and provides important insight into the connection between kindergartners’ health development and later life outcomes. For instance, Duncan et al. (2020) [22] found that positive early childhood development (as measured by the EDI) predicted third-grade academic proficiency in mathematics, English language arts, and literacy among 2,976 kindergarteners in Orange County, California.

The current study utilizes EDI data collected in selected geographic regions across California, one of the most diverse states in the nation, with approximately 40.2 percent of residents identifying as Hispanic/Latino/a, 15.9 percent as Asian, and 6.5 percent as Black [23]. Additionally, California recently made significant investments [24] (over $8 billion) to promote health equity for all children, including initiatives through the California Community Schools Partnership [24], Mental Health Student Services Act [25], Universal Prekindergarten [26], and the Children and Youth Behavioral Health Initiative [27]. These investments must be measured on an ongoing basis to determine the extent to which they are achieving their intended goals. Yet, no statewide, population-level early childhood health development datasets currently exist in California.

We have used the EDI because it represents the most extensive dataset on holistic health development for young children in California, providing a snapshot of kindergartners’ physical health, social-emotional skills, and language and cognitive development. Using 11 years of population-level data on early childhood health development (as measured by the EDI from 2010–2020) we sought to determine the extent to which California kindergarteners (*n* = 106,574) face health development inequities by ethnoracial background and neighborhood-level income.

## Methods

### Procedure

The current study presents an analysis of ethnoracial background, neighborhood-level income, and health development for kindergarten students in California using EDI data, which are maintained at the University of California, Los Angeles. The EDI team established partnerships with local community sites to collect and utilize these data. Within participating school districts, kindergarten teachers receive training on EDI data collection protocols and then report on the five domains of the EDI about each of their students. To ensure a thorough understanding of their students, teachers collect EDI data no earlier than three months after the start of the school year. This timeframe allows them to gather sufficient knowledge about each student before completing the survey. Families were sent a letter to obtain their informed consent and were provided an opt-out option. The EDI requires approximately 10–15 min per child to complete. The teachers then submit their data which is exported to UCLA via a secure server that meets HIPAA standards for security, confidentially, and privacy. All procedures regarding data collection, analysis, and reporting were approved by the UCLA Institutional Review Board.

### Study population

The total California EDI population includes 153,456 children in 71 school districts, and 2,647 census tracts spanning 11 years from 2010 to 2020. Most of the children reside in the counties of Los Angeles, Orange, Fresno, and Alameda. Among the California EDI population, there are 55.7 percent Hispanic/Latino/a students, 19.8 percent white students, 13.4 percent Asian, Native Hawaiian, or other Pacific Islander students, 4.5 percent African American or Black students, and 6.6 percent who are in another ethnoracial category. English Language Learners account for 44.0 percent of students and female students account for 49.9 percent of the population.

### Sample

One of the primary purposes of collecting EDI data is for local communities to identify geographic areas with high levels of health development vulnerability in order to address structural inequities and plan for better resource allocation. Due to the applied nature of these data, the sampling strategy varies from site to site. Some sites only collect data for one year, while others collect data multiple times over several years. Additionally, though the data team strives to collect data from all kindergarten students within a catchment area, this is not always possible. Thus, we employ a series of inclusion and exclusion criteria as well as a probability weight to address this sampling variation.

To minimize bias from selective inclusion of schools or children, we excluded pilot years of data collection, private schools, Head Start programs, observations without probability weights, observations that did not have valid EDI data (at least 75% response on at least four out of five domains), and non-geocoded records. The final study sample includes 106,574 kindergarten students living in 2,472 census tracts and attending 964 schools in 52 school districts throughout California, with data collected between 2010 and 2020. See Additional file 1: Appendix A for the sample flowchart.

To partially account for varying levels of data completeness at the school district level, the study used the National Center for Education Statistics Common Core of Data enrollment figures to compare total number of kindergarten students enrolled in a district in a given year and the total EDI records collected in the same district and year. A probability weight was constructed from this proportion, aiming to increase internal validity by upweighting district-years with higher levels of completeness of EDI data collection and thus lower likelihood of introducing bias into the sample. The probability weight is not intended to address generalizability to the broader California population.

### Measures

#### Early childhood health development

Many studies in the US, Canada, and Australia have employed the EDI to measure early childhood health development, with substantial psychometric investigation. The EDI has undergone multilevel validity, internal consistency, factor structure, and differential item functioning validation [21, 22, 28]. Teachers report on each student in their class, and the data are aggregated to provide reliable measures of children’s health development at a group level or across demographic characteristics (e.g., neighborhood, English learners). Thus, it is a measure of child health development at the population level. EDI data are also linked to students’ home addresses to facilitate place-based analyses.

The EDI includes five domains: 1. Physical health and well-being (13 items); 2. Social competence (26 items); 3. Emotional maturity (30 items); 4. Language and cognitive development (26 items); and 5. Communication skills and general knowledge (8 items). To be “on-track” on a specific domain, a child must be above the 25^th^ percentile of that domain based on the normative US database distribution from the 2008–2009 school year convenience sample (*N* = 10,244). This study examines whether children are on track for each domain separately and on all five domains in total, with dichotomous variables (1 = on-track, 0 = not on-track).

#### Child demographics

Child demographics include ethnoracial background and neighborhood-level ethnoracial-group-specific median household income, since the EDI does not include household-level income. Ethnoracial background was divided into five categories: 1. African American/Black, 2. Asian/Native Hawaiian/Pacific Islander, 3. Hispanic/Latino/a, 4. White, and 5. Other. The ‘other’ ethnoracial category consists of children who are identified by their districts as multiracial, American Indian/Alaskan Native, other, or unknown race/ethnicity). US Census American Community Survey 5-year estimates from the year of the student’s EDI data collection were used to assign ethnoracial-group-specific median household income by census tract.

### Data analysis

First, this study analyzed the data descriptively, presenting ethnoracial-group-specific sample size, household median neighborhood-level income, and percent on-track overall and by domain for the EDI without probability weights. To investigate the extent to which ethnoracial background and neighborhood-level income predict health development, the study utilized a Generalized Estimating Equation (GEE) with the exchangeable correlation structure, clustering at the district-year level, and a log link function to account for dichotomous outcome variables (on-track overall and by domain), representing all coefficients as odds ratios, with a probability weight at the district-year level. GEE-exchangeable is more efficient when calculating within-cluster covariates compared to GEE-independent. Due to the large sample (*n* = 106,574) and cluster size (135 district-years), there are minimal differences in coefficients and interpretation between GEE-exchangeable and GEE-independent. These GEE Models include interaction terms for ethnoracial category by neighborhood-level income as well as by neighborhood-level income-squared to account for the potential for nonlinearity. There are separate models for each domain of the EDI as well as being on-track in all five domains.

Weighted effect coding (WEC) was employed for ethnoracial background rather than dummy variables so that there are no category-specific reference groups. Instead, each ethnoracial group is compared to the weighted mean [29, 30]. WEC is a method of avoiding the common practice of comparing all ethnoracial groups to white individuals as the reference group and has the added benefit of presenting coefficients for all groups within a category [31, 32].

Marginal effects were calculated post-estimation to interpret the combination of interaction terms and quadratic terms and the between-group differences. Marginal estimates were calculated for each ethnoracial group from neighborhood-level incomes of $10,000 to $140,000 in $10,000 increments. Graphs represent this data visually.

There were 340 missing values for ethnoracial background (from the EDI dataset) and 4,955 missing values for neighborhood-level income (from the census-tract-level American Community Survey dataset), making up less than five percent of the total sample. Due to the small amount of missing data, complete case deletion was used to account for missing values.

## Results

Table 1 presents sample size, neighborhood-level median income, and percent on-track by ethnoracial group. Both sample size and neighborhood-level median income vary greatly by ethnoracial group. The majority of students in the sample are Hispanic/Latino/a (56,408 or 52.9%). The lowest proportion of the sample are Black (4,468 or 0.04%). White students have the highest average neighborhood-level median income at $109,676, while Black students have the lowest at $42,631.

**Table 1:** Sample composition and neighborhood income by ethnoracial background

	**Total**	**Asian**	**Black**	**Hispanic**	**White**	**Other**
**N**	106,574	15,014	4,468	56,408	24,356	6,328
**Neighborhood Median Income –** Mean (SD)	$74,623 ($37,867)	$94,884 ($43,254)	$42,631 ($28,255)	$57,404 ($25,671)	$109,676 ($35,297)	$82,004 ($46,883)
**EDI Domains**						
**On-Track Overall**	50.02%	62.08%	37.60%	43.24%	59.87%	52.75%
**Physical Health**	78.69%	87.37%	65.42%	75.76%	82.16%	80.22%
**Social Competence**	76.74%	82.86%	62.02%	74.30%	81.06%	77.80%
**Emotional Maturity**	78.89%	82.88%	64.96%	77.73%	81.57%	79.39%
**Language & Cognition**	70.55%	83.78%	65.06%	62.51%	81.04%	74.39%
**General Knowledge**	73.82%	77.35%	72.22%	67.98%	84.45%	77.65%

Unweighted. Neighborhood Income is the race/ethnicity-specific household median income based on the US Census American Community Survey 5-year estimates applied to the student’s year of EDI data collection (ranging from 2010–2020). Income Quintile 1 is the lowest, and 5 is the highest

Table 2 presents the results from the GEE-exchangeable models of health development by ethnoracial background and neighborhood-level income for on-track on all five domains as well as each of the five domains separately. Ethnoracial background is interacted with neighborhood-level income and neighborhood-level income-squared due to the suggestion of non-linear trajectories in preliminary analyses. The model reinforces the potential of non-linearity, since at least one quadratic term coefficient is significant at the *p* < 0.05 level in each of the models.

**Table 2:** Generalized estimation equation of association of health development of kindergartners with neighborhood income and ethnoracial background including interaction and quadratic term

	(Model 1)	(Model 2)	(Model 3)	(Model 4)	(Model 5)	(Model 6)
	**On Track Overall**	**Physical Health**	**Emotional Maturity**	**Social Competence**	**Language and Cognition**	**General Knowledge**
**Ethnoracial Background**						
**Asian**	**1.574**	**1.737**	1.140	1.223	**1.704**	0.914
	**(1.297—1.911)**	**(1.355—2.228)**	(0.894—1.454)	(0.984—1.522)	**(1.355—2.142)**	(0.752—1.111)
**Black**	**0.688**	**0.548**	**0.717**	**0.591**	1.010	1.102
	**(0.543—0.871)**	**(0.453—0.663)**	**(0.587—0.876)**	**(0.493—0.708)**	(0.779—1.311)	(0.914—1.330)
**Hispanic**	0.960	1.076	**1.160**	**1.206**	**0.815**	0.909
	(0.854—1.079)	(0.962—1.203)	**(1.043—1.289)**	**(1.081—1.346)**	**(0.706—0.940)**	(0.801—1.032)
**White**	0.800	**0.671**	**0.673**	**0.617**	1.004	1.168
	(0.638—1.005)	**(0.515—0.874)**	**(0.535—0.846)**	**(0.494—0.772)**	(0.756—1.334)	(0.893—1.528)
**Other**	**1.439**	1.027	1.144	1.117	**1.525**	**1.409**
	**(1.163—1.779)**	(0.790—1.335)	(0.930—1.407)	(0.877—1.423)	**(1.111—2.094)**	**(1.093—1.817)**
**Median Income**	**1.078**	**1.055**	**1.051**	**1.063**	**1.083**	**1.077**
	**(1.056—1.100)**	**(1.028—1.084)**	**(1.025—1.077)**	**(1.041—1.086)**	**(1.059—1.109)**	**(1.052—1.103)**
**Median Income**^**2**^	**0.998**	0.999	0.999	**0.998**	0.999	0.999
	**(0.997—0.999)**	(0.998—1.000)	(0.998—1.000)	**(0.998—0.999)**	(0.998—1.000)	(0.997—1.000)
**Interaction: Ethnoracial Background and Median Income**						
**Asian**	1.000	0.997	1.024	1.032	1.028	**1.038**
	(0.967—1.034)	(0.953—1.044)	(0.978—1.071)	(0.993—1.073)	(0.982—1.076)	**(1.000—1.078)**
**Black**	1.012	1.037	**0.929**	1.007	0.981	0.990
	(0.954—1.074)	(0.981—1.096)	**(0.870—0.992)**	(0.953—1.064)	(0.927—1.038)	(0.940—1.044)
**Hispanic**	**0.973**	**0.967**	**0.976**	**0.954**	0.978	**0.971**
	**(0.954—0.994)**	**(0.946—0.988)**	**(0.956—0.997)**	**(0.933—0.975)**	(0.953—1.003)	**(0.948—0.995)**
**White**	**1.077**	**1.073**	**1.067**	**1.098**	**1.053**	**1.058**
	**(1.037—1.119)**	**(1.024—1.124)**	**(1.024—1.112)**	**(1.056—1.141)**	**(1.001—1.108)**	**(1.008—1.110)**
**Other**	**0.955**	1.008	0.978	0.986	0.960	0.968
	**(0.918—0.994)**	(0.961—1.058)	(0.940—1.018)	(0.944—1.030)	(0.907—1.017)	(0.923—1.016)
**Interaction: Ethnoracial Background and Median Income Squared**						
**Asian**	1.000	1.000	0.999	0.999	0.999	0.999
	(0.999—1.002)	(0.998—1.002)	(0.997—1.001)	(0.997—1.001)	(0.997—1.001)	(0.997—1.001)
**Black**	0.999	0.998	**1.004**	0.999	1.000	0.999
	(0.997—1.002)	(0.995—1.001)	**(1.000—1.007)**	(0.996—1.002)	(0.997—1.002)	(0.997—1.002)
**Hispanic**	**1.001**	**1.001**	**1.001**	**1.002**	**1.001**	**1.001**
	**(1.000—1.002)**	**(1.000—1.002)**	**(1.000—1.002)**	**(1.001—1.003)**	**(1.000—1.002)**	**(1.000—1.002)**
**White**	**0.997**	0.998	0.998	**0.997**	0.998	**0.997**
	**(0.996—0.999)**	(0.996—1.000)	(0.996—1.000)	**(0.995—0.998)**	(0.996—1.000)	**(0.995—0.999)**
**Other**	**1.002**	1.000	1.001	1.001	1.001	1.001
	**(1.000—1.003)**	(0.998—1.002)	(0.999—1.002)	(0.999—1.002)	(0.999—1.004)	(0.999—1.003)
**Constant**	**0.622**	**2.680**	**2.771**	**2.285**	**1.522**	**1.795**
	**(0.553—0.699)**	**(2.320—3.096)**	**(2.430—3.159)**	**(2.024—2.580)**	**(1.329—1.744)**	**(1.588—2.030)**

We utilize Generalized Estimating Equation (GEE) with the exchangeable correlation structure, clustering at the district-year level, and a log link function to account for the dichotomous outcome variables. Each column represents a distinct model with ‘on-track’ on all domains or one of the EDI domains (physical health, emotional maturity, social competence, language and cognition, general knowledge) as the outcome variable. The models include a probability weight to account for varied sample completeness by district-year. All coefficients are represented as odds ratios. Weighted effect coding was used instead of a reference group so estimates are compared to the weighted average. Child *N* = 106,574, district-year cluster *N* = 132. The unit of analysis is the child. All outcomes are dichotomous (i.e., 1 = on-track, 0 = not on-track). Bolded estimates are significant at the *p* < 0.05 level

**Fig. 1:**
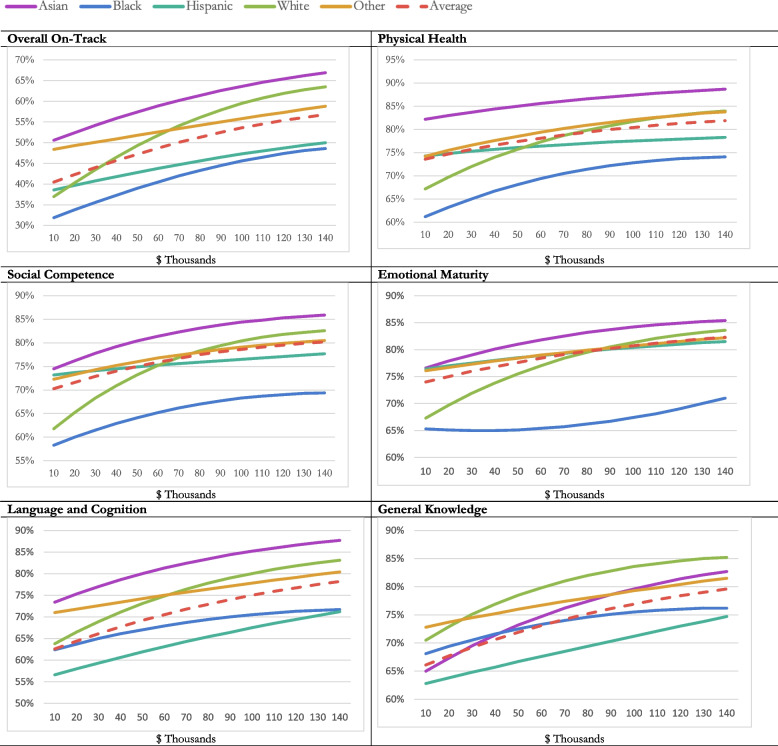
Marginal Plot of Percent Overall On-Track and Five Domains of Health Development by Neighborhood Income for Different Ethnoracial Backgrounds. Notes: Output based on marginal post-estimation commands from model in Table 2. Marginal estimates were calculated for each ethnoracial group from neighborhood incomes of $!0,000 to $140,000 in $10,000 increments. Y-axis represents the estimated percent of students projected to be on-track on all five domains of developmental health using the EDI

The models in Table 2 are graphically represented in Fig. 1 using marginal post-estimation commands, calculating estimated percentage of students on-track in health development from $10,000 to $140,000 using increments of $10,000. Each line represents an ethnoracial group except for the red dotted line, which represents the average (weighted by ethnoracial group). For overall on-track, while the likelihood of being on-track increases with neighborhood-level income for all ethnoracial groups, the slope of the lines vary. The strongest association between neighborhood-level income and health development is seen in white students, with a 27 percent increase in likelihood of being on-track from a neighborhood-level household income of $10,000 to $140,000. Asian and Black students have similar slopes—increasing 16 and 17 percent across neighborhood income levels respectively. Hispanic/Latino/a students see an increase of 11 percent and students in the Other group see an increase of 10 percent. Though the slopes are similar for Black and Asian students, their overall likelihood of being on-track differs widely. At all neighborhood income levels, Asian students are most likely to be on-track in all EDI domains. Black students are least likely to be on-track. In fact, Black students in the highest neighborhood income level of $140,000 are still less likely to be on-track (49%) than Asian students in the lowest neighborhood income quintile of $10,000 (51%). These patterns are also somewhat evident in the physical health, emotional maturity, and social competence models. See Additional file 2: Appendix B for marginal postestimation graphs representing the confidence intervals across neighborhood-level income groups for each ethnoracial group compared to the weighted average, which shows that for all of those except emotional maturity, trends for Asian and Black students are statistically different than the weighted average (*p* < 0.05) at each neighborhood income level. For emotional maturity, the trend for Black students is not significant. For language and cognition, Hispanic/Latino/a students have the lowest on-track likelihood across neighborhood income levels. The trends for Asian and Hispanic/Latino/a students are statistically significant at all neighborhood income levels (*p* < 0.05). For general knowledge, white students have the highest likelihood of being on-track after $30,000 neighborhood-level median income, and Hispanic/Latino/a students have the lowest likelihood. The trend for Hispanic/Latino/a students is significant at all neighborhood income levels, and that of white students is significant from $20,000 on.

## Discussion

This study addresses an important gap in the literature by examining population-level kindergartners’ (*n*= 106,574) health development across domains, stratified by ethnoracial background and neighborhood-level income. While income has long been established as a significant predictor of health outcomes across the lifespan [33, 34], including school readiness [11–13], these data support findings that ethnoracial background plays a significant role in the developmental origins of inequity beyond income. For instance, for total kindergartners who are on-track using the EDI, even the poorest Asian children experience better health development outcomes than the most affluent Black or Hispanic/Latino/a children in our sample. Black children experience worse health development outcomes across the full neighborhood-level income gradient [35]. These results suggest that structural racism may impact health even in the earliest years, and reinforces the minorities' diminishing return theory, which suggests that income does not lead to substantial health gains for minoritized groups as it does for whites [36].

Our data also detected population-based resilience. For instance, for total kindergartners who are on-track using the EDI, Hispanic/Latino/a children in the lowest-income neighborhoods have higher levels of health development, on average, than their Black and white counterparts. Black and Hispanic/Latino/a children in wealthier neighborhoods have higher levels of health development on average than their lower income counterparts, but the slopes for both groups are not nearly as steep as the income-health development slope for white children. Compared to all other ethnoracial populations, white children have the steepest gradient in total health development, suggesting that their outcomes are most tied to income. This also suggests that working class white children occupy a social position more closely linked with their working-class Black and Hispanic/Latino/a peers than they do with affluent white children.

The five domains of health development offer additional nuance. Physical health and social competence follow a pattern most similar to that of overall on-track. For emotional maturity, at income levels higher than around $80,000, all ethnoracial groups have similar levels of emotional maturity except Black students, whose likelihood of being on-track is around 10 percentage points lower than the weighted average across income groups and only increases six percentage points from the lowest to the highest income level. General knowledge does not follow a similar pattern – white students mostly have the highest on-track likelihoods while Hispanic/Latino/a students have the lowest. In further research, these patterns may provide insight into the mechanisms by which structural inequities contribute to health development.

The study’s findings represent a collective snapshot – an outcome – of kindergartners experiences within their respective ecosystems during the first five years of life. Yet, the precise reasons behind the differences observed between health development domains remains unknown. For instance, kindergarten populations exhibit healthier physical health and emotional maturity compared to language and cognitive development. Notably, all data predates the COVID-19 pandemic era, and recent studies have found heightened disparities in school readiness and young children’s development during this period [37, 38]. Sustained, long-term supports are essential to counteract the negative impacts of the pandemic, particularly the exacerbated social inequities and disparities [39]. Further research is crucial to investigate the key factors within a child’s ecosystem, especially during the COVID-19 pandemic era, which may underpin the variations identified in the present study.

### Limitations

This study has several limitations that need to be acknowledged. First, our sample was a convenience sample from selected sites that collected EDI data. It is thus not representative of the entire state of California. However, it is worth noting that the sample size included over 100,000 kindergartners. This sample is also only of public school students, excluding those who attend private school. Additionally, districts varied widely in completeness of data collection, though the intention in most districts was to collect data from all kindergartners in a given year or sampling cycle (up to three years). To partially address this issue post-hoc, we employed a probability weight based on total enrollment of kindergartners, upweighting districts with higher levels of completeness. Second, while we have data ranging from 2010–2020, the number of years of data per school district varies widely, so it was not possible to investigate the data longitudinally. Thus, we collapsed all years of data into one cross-sectional sample. Third, due to the unavailability of individual family income data, we had to rely on median household income from the census tract. While these estimates offer a population-level perspective at the neighborhood level, they may provide a better measure of the local resource context. To better approach an estimate of household income, we used neighborhood income estimates specific to the child’s ethnoracial background. Fourth, the study used five broad categories for ethnoracial background. The use of these categories enhances comparability and interpretability of our findings, given their resemblance to widely used classifications. The ‘other’ ethnoracial category does not represent a specific or cohesive population, so we cannot draw meaningful conclusions for this category. Future research should aim to further disaggregate ethnoracial groups, including understudied populations such as Native Americans. Finally, due to data limitations, we were unable to assess potential confounding factors such as parental mental health, education, marital status, employment, and other positive and negative influences. Consequently, further research is necessary to address these gaps.

## Conclusions and implications

Life course health development studies clearly indicate that early inequities have long-term consequences, leading to exacerbated and severe health inequities in later stages of life. It is thus imperative to identify investments that can effectively and efficiently address health inequities as soon as possible. California’s recent early childhood investments present an extraordinary opportunity for the state to take bold action in addressing these glaring health development inequities. An important initial step is to utilize a sufficiently sensitive population-level measure such as the EDI to serve as a monitoring and accountability system that can illuminate the landscape of inequities and progress across California. This data system can then inform the design, implementation, and evaluation of place-based child health initiatives, allowing local leaders to tailor interventions based on the specific needs of their communities.

A valid life course population measurement also provides a critical advancement by illuminating the health or vulnerability of ecosystems. Since government investments often prioritize a service sector framework, communities face the challenge of effectively braiding and bridging siloed funding streams and services. Synthesizing life course theory with population measurement allows stakeholders (e.g., practitioners, policymakers, researchers, and local community leadership) to focus on the holistic well-being of a dynamic and adaptive ecosystem. By employing such an approach, data systems present new opportunities to embolden and mobilize stakeholders, enabling them to recognize the foundational and historical patterns of resilience within an ecosystem, thereby fostering widespread success and nurturing human flourishing.

Adopting a life course population health measurement also serves as an added benefit of bringing together various stakeholders, including local governments, philanthropy organizations, local education agencies, and schools. By prioritizing targeted, place-based interventions instead of simply offering a universal, one-size-fits-all approach, this strategy enables all communities to collaborate towards a shared goal, without perpetuating advantages for privileged groups [40]. Aiming to have all kindergartners on-track on all five domains of health development can serve as a universal goal for community and government leaders. They can then create targeted interventions based on the specific needs of neighborhoods and communities, ensuring that marginalized groups receive the necessary support to achieve optimal health outcomes.

Targeted universal programs for healthy development require data systems that can be used to set universal goals for health promotion. Traditionally, California’s state health surveillance systems have primarily focused on traditional public health indicators over multidimensional measures of health and well-being. While the state’s educational data has made significant improvements (i.e., the Healthy Kids Survey), these data have been primarily reported as a school measure and not as a robust ecosystem measure [41]. The recent investment funds present an opportunity for transformational systems change that elevates a whole child equity measurement system. Such a longitudinal population data infrastructure would allow the State of California to investigate and challenge the structural and historical inequities which lead to the health disparities shown in this study. Ultimately, having a state-wide, life course population data system can drive systems transformation, facilitate shared learning and accountability, enhance multisector collaboration, and empower community engagement and co-ownership of shared problems and shared solutions.

### Supplementary Information

**Additional file 1: Appendix A:** Sample Flowchart.

**Additional file 2: Appendix B:** Marginal Post-Estimation Graphs Representing the Confidence Intervals Across Income Groups by Ethnoracial Group Compared to the Weighted Average.

## Data Availability

The datasets used and analyzed during the current study are not publicly available due to contractual obligations with the developer but are available from the corresponding author on reasonable request.

## Footnotes

1 Throughout this article, we capitalize “Black” but not “white” as descriptors of racial groups. Black represents a group of people that shares many histories and cultures, while white represents a social construct stemming chiefly from colonization, oppression, and power, more as a negation of other groups of people than a descriptor of a group with shared experiences and culture.^9.^

## References

[1] Halfon N, Larson K, Lu M, Tullis E, Russ S (2014). Lifecourse health development: past, present and future. Matern Child Health J.

[2] Halfon N, Forrest CB, Lerner RM, Faustman EM, eds. Handbook of Life Course Health Development. New York, NY: Springer International Publishing; 2018. 10.1007/978-3-319-47143-3. 31314220

[3] Hertzman C, Boyce T (2010). How experience gets under the skin to create gradients in developmental health. Annu Rev Public Health..

[4] Halfon N, Hochstein M (2002). Life Course Health Development: An Integrated Framework for Developing Health, Policy, and Research. Milbank Q.

[5] Halfon N, Russ SA, Schor EL (2022). The emergence of life course intervention research: optimizing health development and child well-being. Pediatrics.

[6] World Health Organization. The Implications for Training of Embracing: A Life Course Approach to Health. World Health Organization; 2000. https://apps.who.int/iris/handle/10665/69400. Accessed 10 Nov 2022.

[7] Marmot M (2005). Social determinants of health inequalities. Lancet Lond Engl.

[8] Priest N, Paradies Y, Trenerry B, Truong M, Karlsen S, Kelly Y (1982). A systematic review of studies examining the relationship between reported racism and health and wellbeing for children and young people. Soc Sci Med.

[9] Margolin G, Gordis EB (2000). The effects of family and community violence on children. Annu Rev Psychol.

[10] Duncan GJ, Murnane RJ. Whither Opportunity? Russell Sage Foundation; 2011. http://www.jstor.org/stable/10.7758/9781610447515. Accessed 10 Nov 2022.

[11] Morrissey TW, Vinopal KM (2018). Neighborhood disadvantage and children’s academic skills and behavior in early elementary school. J Marriage Fam.

[12] Vinopal K, Morrissey TW (2020). Neighborhood disadvantage and children’s cognitive skill trajectories. Child Youth Serv Rev.

[13] Wolf S, Magnuson KA, Kimbro RT (2017). Family poverty and neighborhood poverty: Links with children’s school readiness before and after the Great Recession. Child Youth Serv Rev.

[14] Johnson J, Perrigo JL, Deavenport-Saman A (2021). Effect of home environment on academic achievement in child protective service-involved children: results from the second national survey of child and adolescent well-being study. Child Abuse Negl.

[15] Miller P, Votruba-Drzal E (2013). Early academic skills and childhood experiences across the urban–rural continuum. Early Child Res Q.

[16] Nelson RF (2005). The impact of ready environments on achievement in kindergarten. J Res Child Educ.

[17] Campbell FA, Pungello EP, Burchinal M (2012). Adult outcomes as a function of an early childhood educational program: an abecedarian project follow-up. Dev Psychol.

[18] Campbell FA, Ramey CT, Pungello E, Sparling J, Miller-Johnson S (2002). Early childhood education: young adult outcomes from the abecedarian project. Appl Dev Sci.

[19] Fitzpatrick C, Boers E, Pagani LS (2020). Kindergarten Readiness, Later Health, and Social Costs. Pediatrics.

[20] Shonkoff JP, Boyce WT, McEwen BS (2009). Neuroscience, molecular biology, and the childhood roots of health disparities: building a new framework for health promotion and disease prevention. JAMA.

[21] Janus M, Offord DR (2007). Development and psychometric properties of the Early Development Instrument (EDI): A measure of children’s school readiness. Can J Behav Sci Rev Can Sci Comport.

[22] Duncan RJ, Duncan GJ, Stanley L, Aguilar E, Halfon N (2020). The Kindergarten Early Development Instrument Predicts Third Grade Academic Proficiency. Early Child Res Q.

[23] U.S. Census Bureau. 2021 American Community Survey 1-Year Estimates Subject Tables. 2022. https://data.census.gov/table?q=age+&g=0400000US06&tid=ACSST1Y2021.S0101. Accessed 4 Nov 2022.

[24] Governor Newsom Unveils New Plan to Transform Kids’ Mental Health. California Governor. https://www.gov.ca.gov/2022/08/18/governor-newsom-unveils-new-plan-to-transform-kids-mental-health/. Published August 18, 2022. Accessed 10 Nov 2022.

[25] California Mental Health Services Oversight and Accountability Commission. School Mental Health. MHSOAC. https://mhsoac.ca.gov/initiatives/school-mental-health/. Published November 22, 2020. Accessed 22 Nov 2022.

[26] D’Souza K. How California’s new universal transitional kindergarten program will be rolled out. EdSource. https://edsource.org/2021/how-californias-new-universal-transitional-kindergarten-program-will-be-rolled-out/657818. Published July 12, 2021. Accessed 10 Nov 2022.

[27] California Health and Human Services Agency. Children and Youth Behavioral Health Initiative. California Health and Human Services. https://www.chhs.ca.gov/home/children-and-youth-behavioral-health-initiative/. Published 2022. Accessed 10 Nov 2022.

[28] Janus M, Brinkman SA, Duku EK (2011). Validity and Psychometric Properties of the Early Development Instrument in Canada, Australia, United States, and Jamaica. Soc Indic Res.

[29] Nieuwenhuis R, te Grotenhuis M, Pelzer B (2017). Weighted Effect Coding for Observational Data with wec. R J.

[30] Te Grotenhuis M, Pelzer B, Eisinga R, Nieuwenhuis R, Schmidt-Catran A, Konig R (2016). When size matters: advantages of weighted effect coding in observational studies. Int J Public Health.

[31] Mayhew MJ, Simonoff JS (2015). Non-White, No More: Effect Coding as an Alternative to Dummy Coding with Implications for Higher Education Researchers. J Coll Stud Dev.

[32] Ro HK, Bergom I (2020). Expanding Our Methodological Toolkit: Effect Coding in Critical Quantitative Studies. New Dir Stud Serv.

[33] Boyce WT, Riolo RL, Suomi SJ (2017). Developmental Complexity: Modeling Social Inequalities in Young Children and Macaques. Growing Inequality: Bridging Complex Systems, Population Health, and Disparities.

[34] Ostrove JM, Adler NE, Kuppermann M, Washington AE (2000). Objective and subjective assessments of socioeconomic status and their relationship to self-rated health in an ethnically diverse sample of pregnant women. Health Psychol Off J Div Health Psychol Am Psychol Assoc.

[35] Bailey ZD, Krieger N, Agénor M, Graves J, Linos N, Bassett MT (2017). Structural racism and health inequities in the USA: evidence and interventions. Lancet Lond Engl.

[36] Assari S, Lapeyrouse LM, Neighbors HW (2018). Income and Self-Rated Mental Health: Diminished Returns for High Income Black Americans. Behav Sci.

[37] González M, Loose T, Liz M (2022). School readiness losses during the COVID-19 outbreak. A comparison of two cohorts of young children. Child Dev..

[38] Penna A, de Aquino CM, Pinheiro MSN (2023). Impact of the COVID-19 pandemic on maternal mental health, early childhood development, and parental practices. BMC Public Health.

[39] Perrigo JL, Samak A, Hurlburt M (2022). Minority and low-SES families’ experiences during the early phases of the COVID-19 pandemic crises. A qualitative study. Child Youth Serv Rev..

[40] powell john, Ake W, Menendian S. Targeted Universalism: Policy & Practice. Haas Institute for a Fair and Inclusive Society: University of California, Berkeley; 2019. https://belonging.berkeley.edu/targeted-universalism. Accessed 19 Oct 2022.

[41] California Department of Education. California Healthy Kids Survey. https://www.cde.ca.gov/ls/he/at/chks.asp. Published March 10, 2022. Accessed 9 Dec 2022.

