# Supplementary material for: Income is not an equalizer: health development inequities by ethnoracial backgrounds in California kindergartners
Source: BMC Public Health. 2023 Dec 11;23:2474. doi: 10.1186/s12889-023-17246-7 (PMC10714585; doi:10.1186/s12889-023-17246-7)
Supplement: Supplementary file 1 — Additional file 1: Appendix A: Sample Flowchart. [file 12889_2023_17246_MOESM1_ESM.docx]

**Appendix A: Sample Flowchart**

Exclude non-geocoded observations: Districts=52 ; Census tracts=2,537 ; Students=111,632

Starting sample: Districts=71; Census tracts= 2,647; Students=153,456

Exclude invalid observations: Districts=52; Census tracts=2,537; Students=112,981

Exclude observations without probability weight: Districts=52; Census tracts= 2,583; Students=121,184

Exclude Head Start and Private Schools: Districts=67; Census tracts=2,606; Students=133,818

Exclude pilot years: Districts=70; Census tracts= 2,608; Students=134,123

Complete Case Deletion: Districts= 52; Census tracts=2,474; Students=106,574
