# Supplementary material for: Income is not an equalizer: health development inequities by ethnoracial backgrounds in California kindergartners
Source: BMC Public Health. 2023 Dec 11;23:2474. doi: 10.1186/s12889-023-17246-7 (PMC10714585; doi:10.1186/s12889-023-17246-7)
Supplement: Supplementary file 2 — Additional file 2: Appendix B: Marginal Post-Estimation Graphs Representing the Confidence Intervals Across Income Groups by Ethnoracial Group Compared to the Weighted Average. [file 12889_2023_17246_MOESM2_ESM.docx]

**Appendix B: Marginal Post-Estimation Graphs Representing the Confidence Intervals Across Income Groups by Ethnoracial Group Compared to the Weighted Average**

**On Track Overall**

| **Asian** | **Black** |
| --- | --- |
| 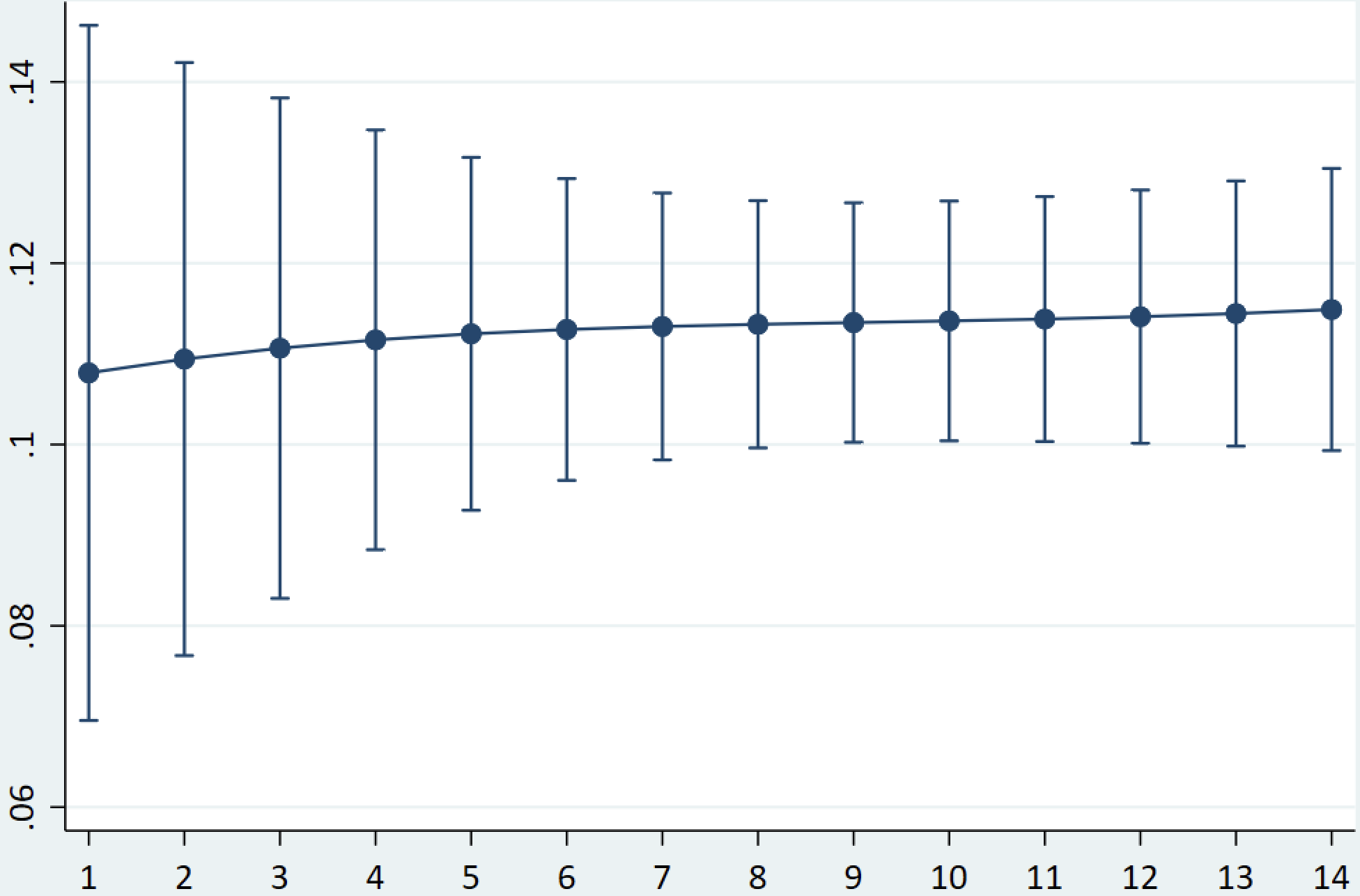 | 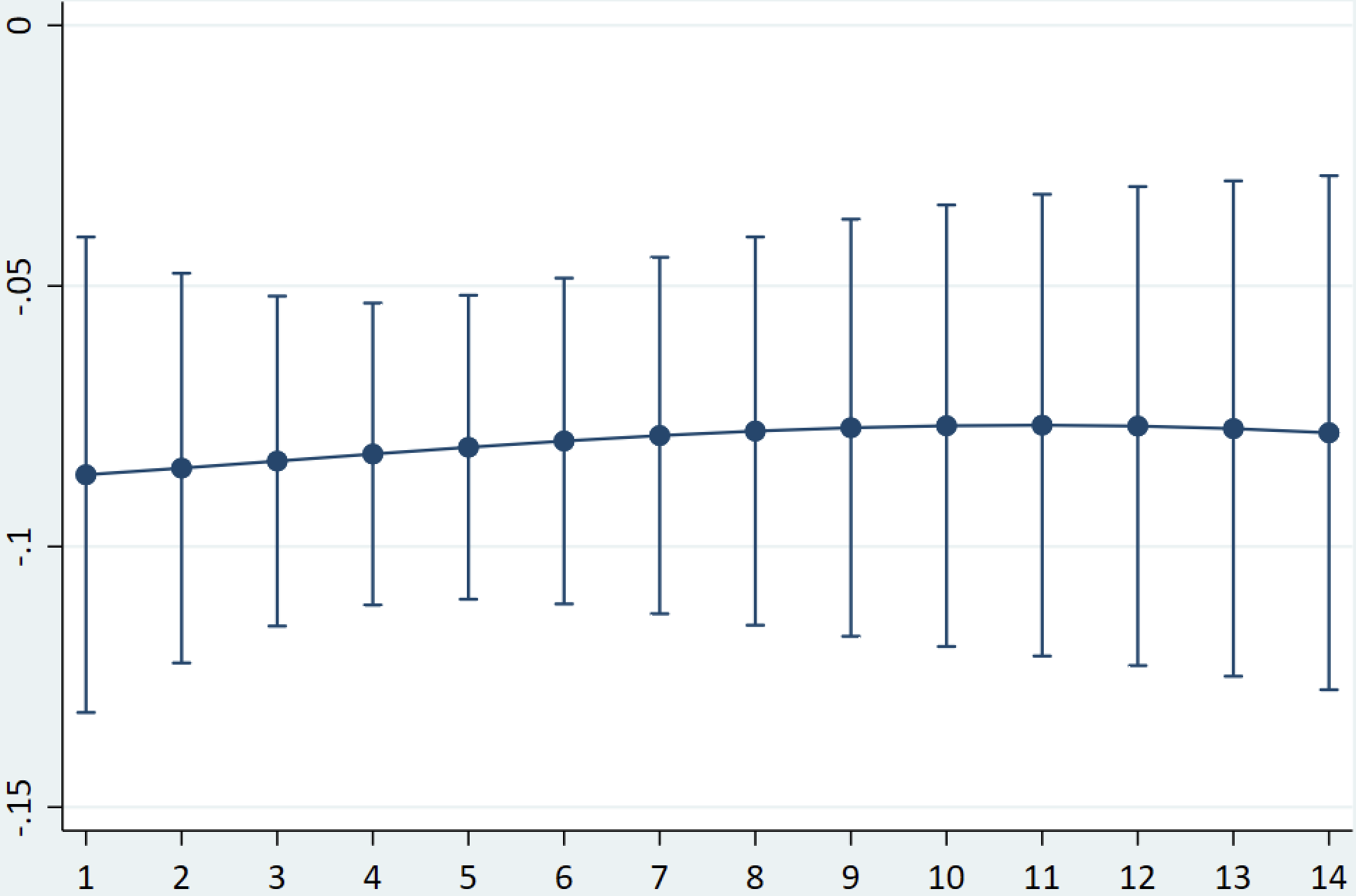 |
| Hispanic | White |
| 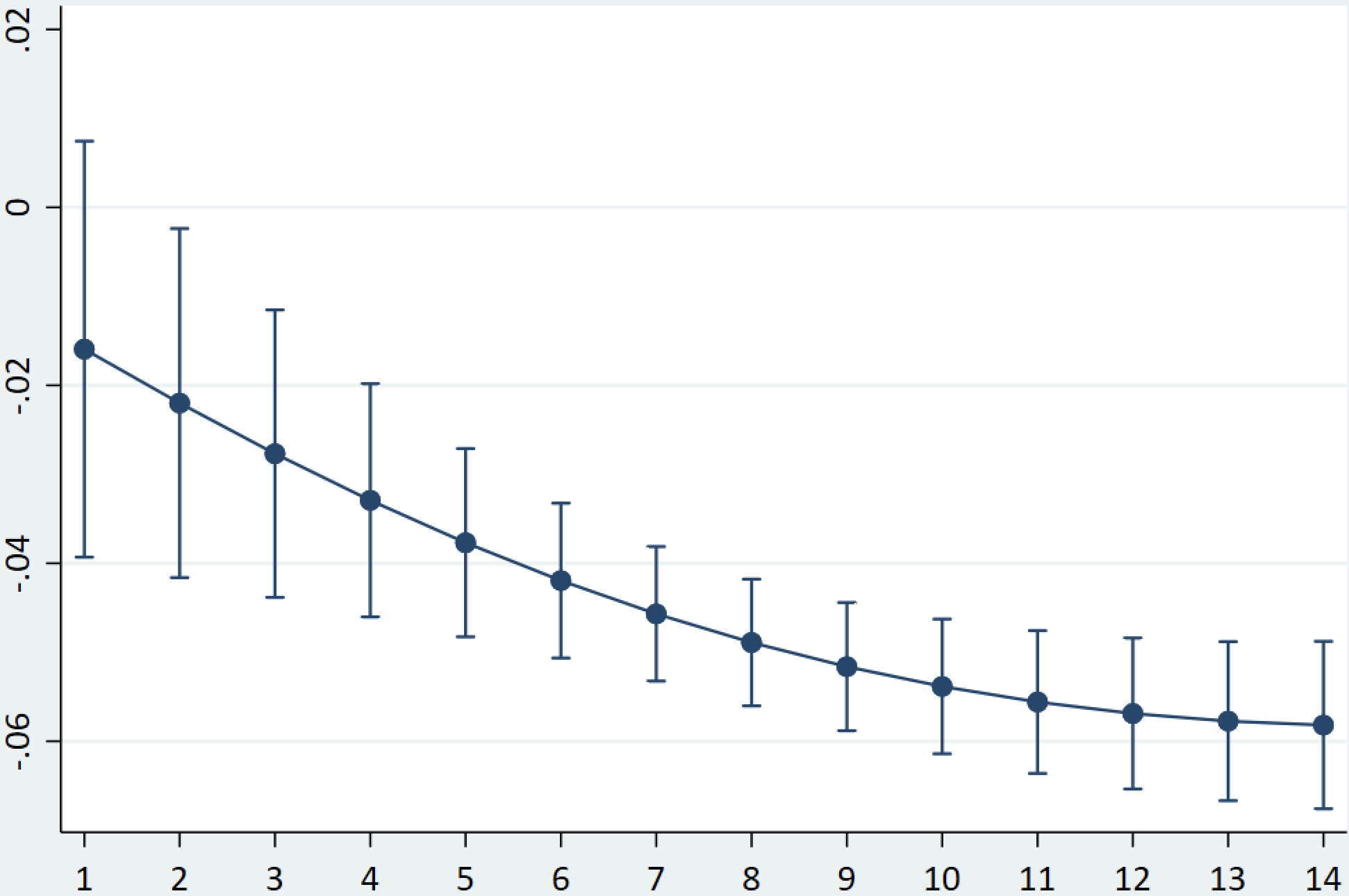 | 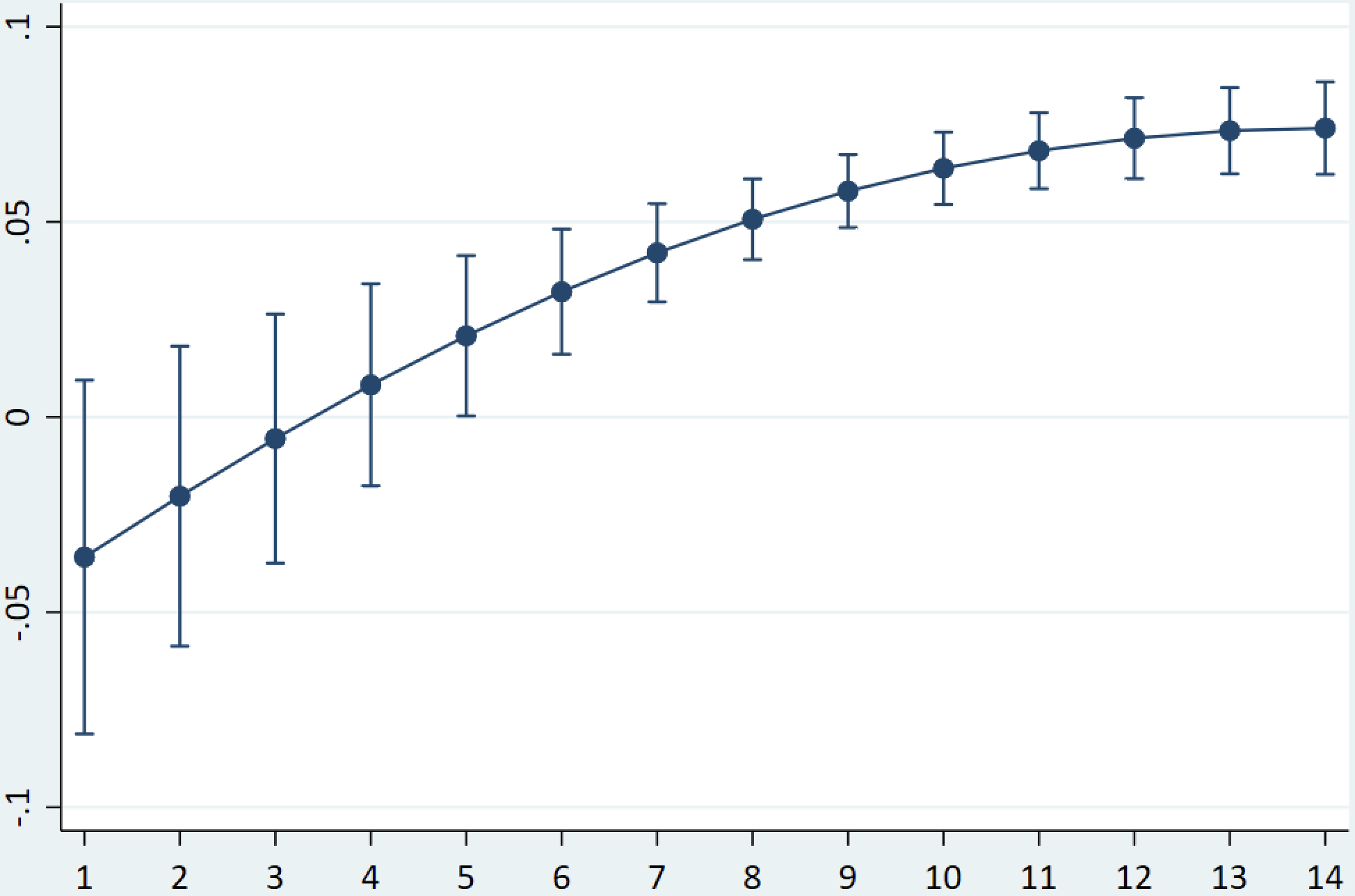 |
| Other |  |
| 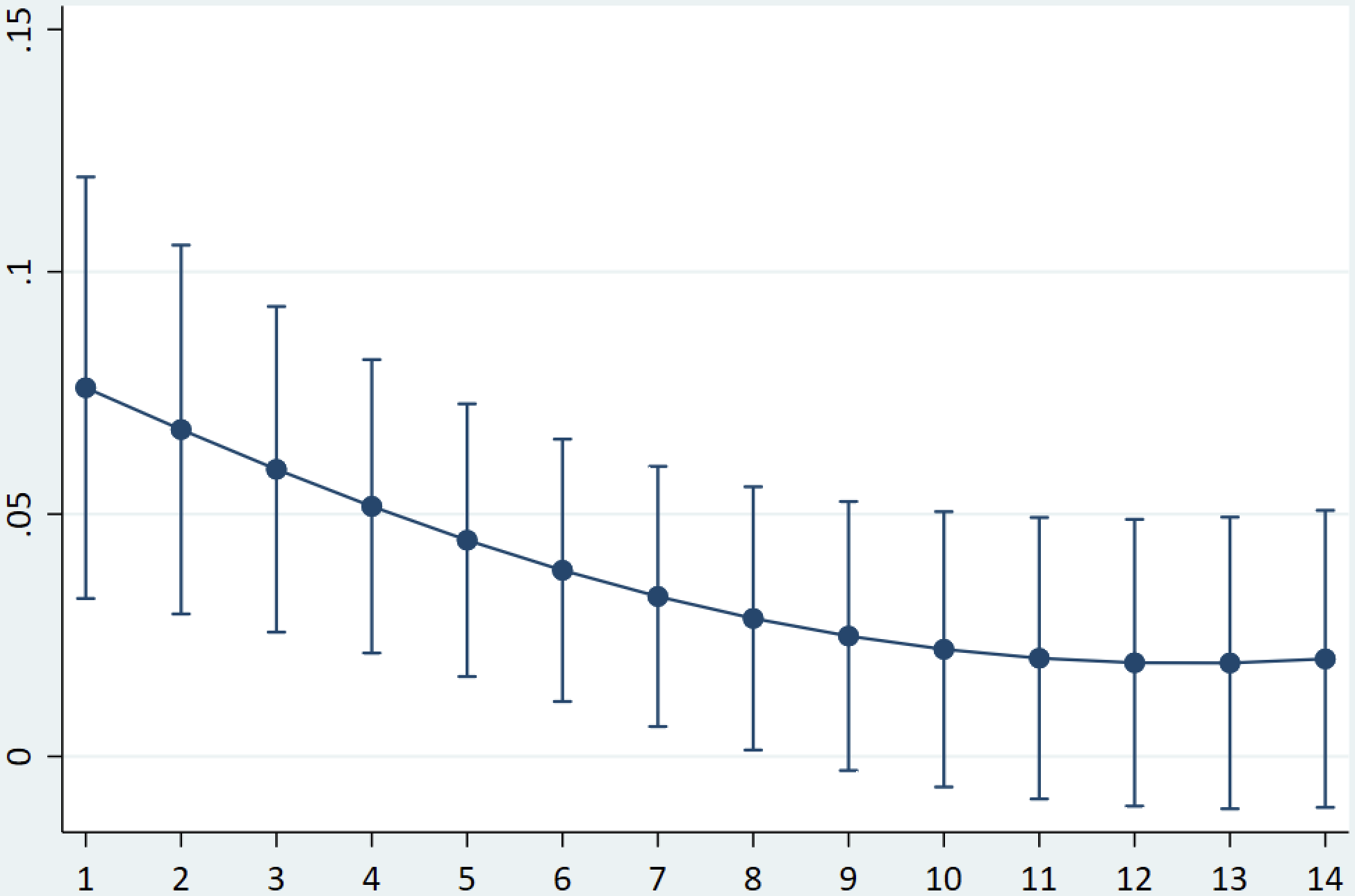 |  |

Notes: X-axis represents dollars in ten thousands.

**Physical Health**

| **Asian** | **Black** |
| --- | --- |
| 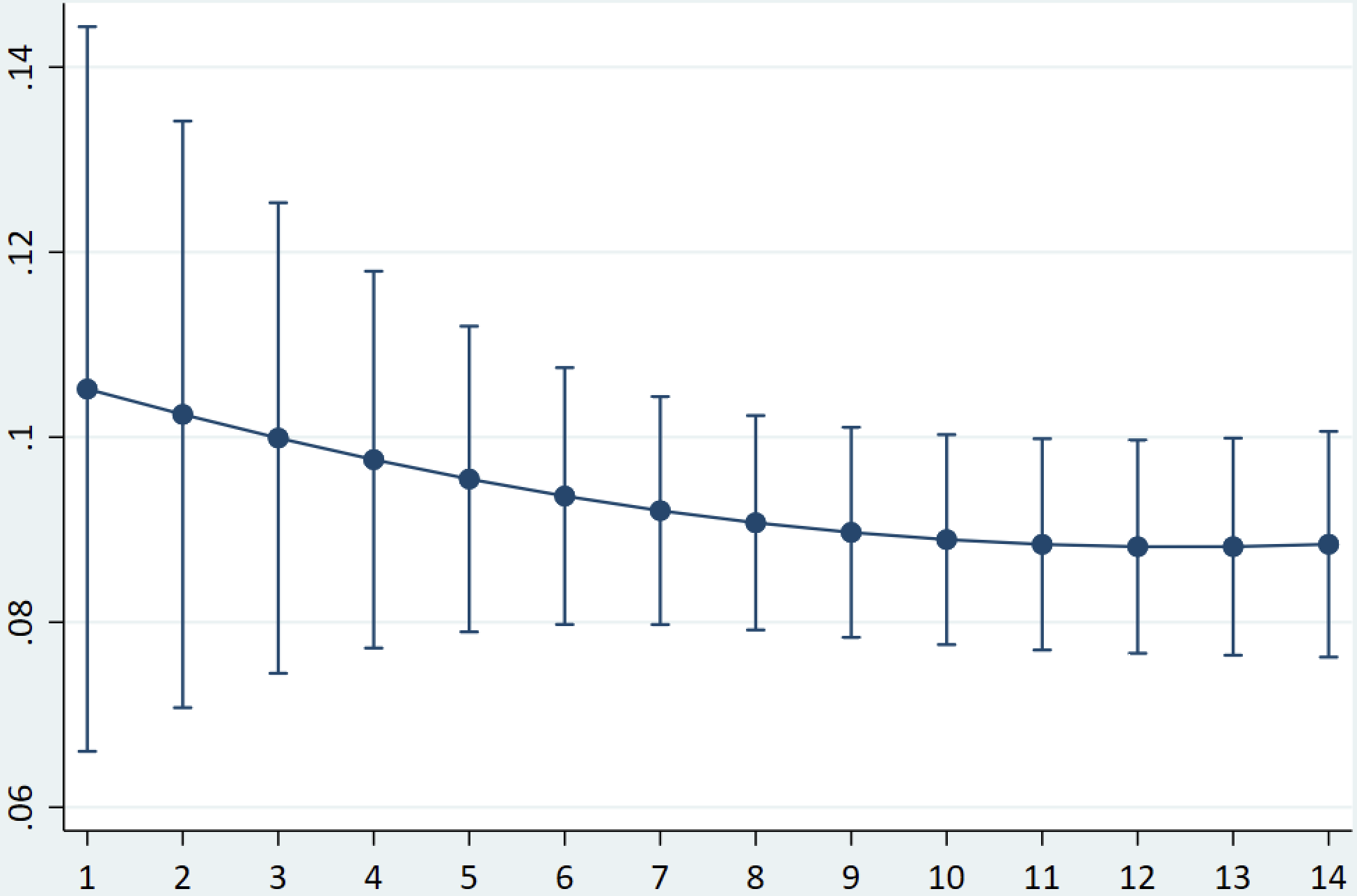 | 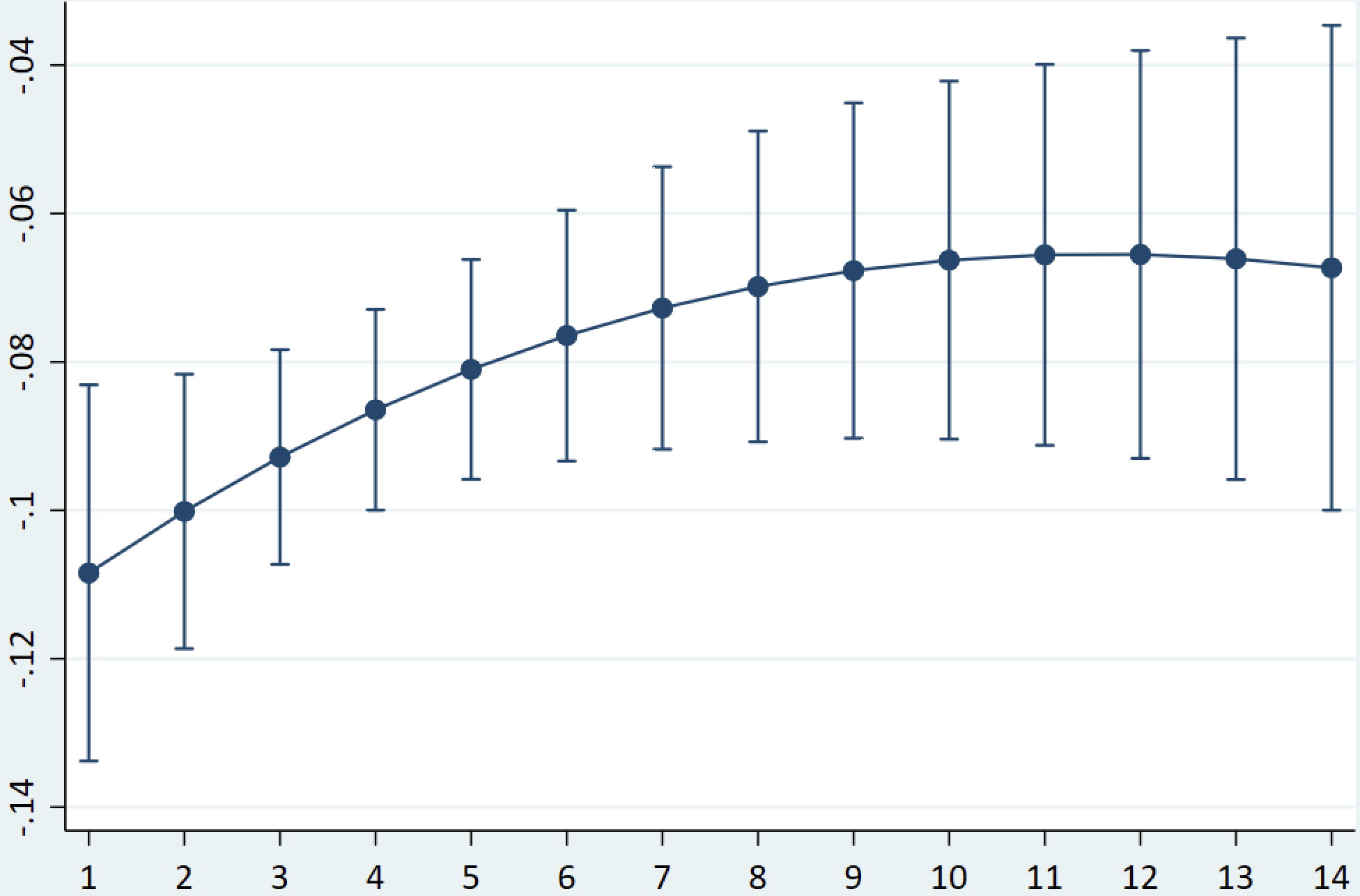 |
| Hispanic | White |
| 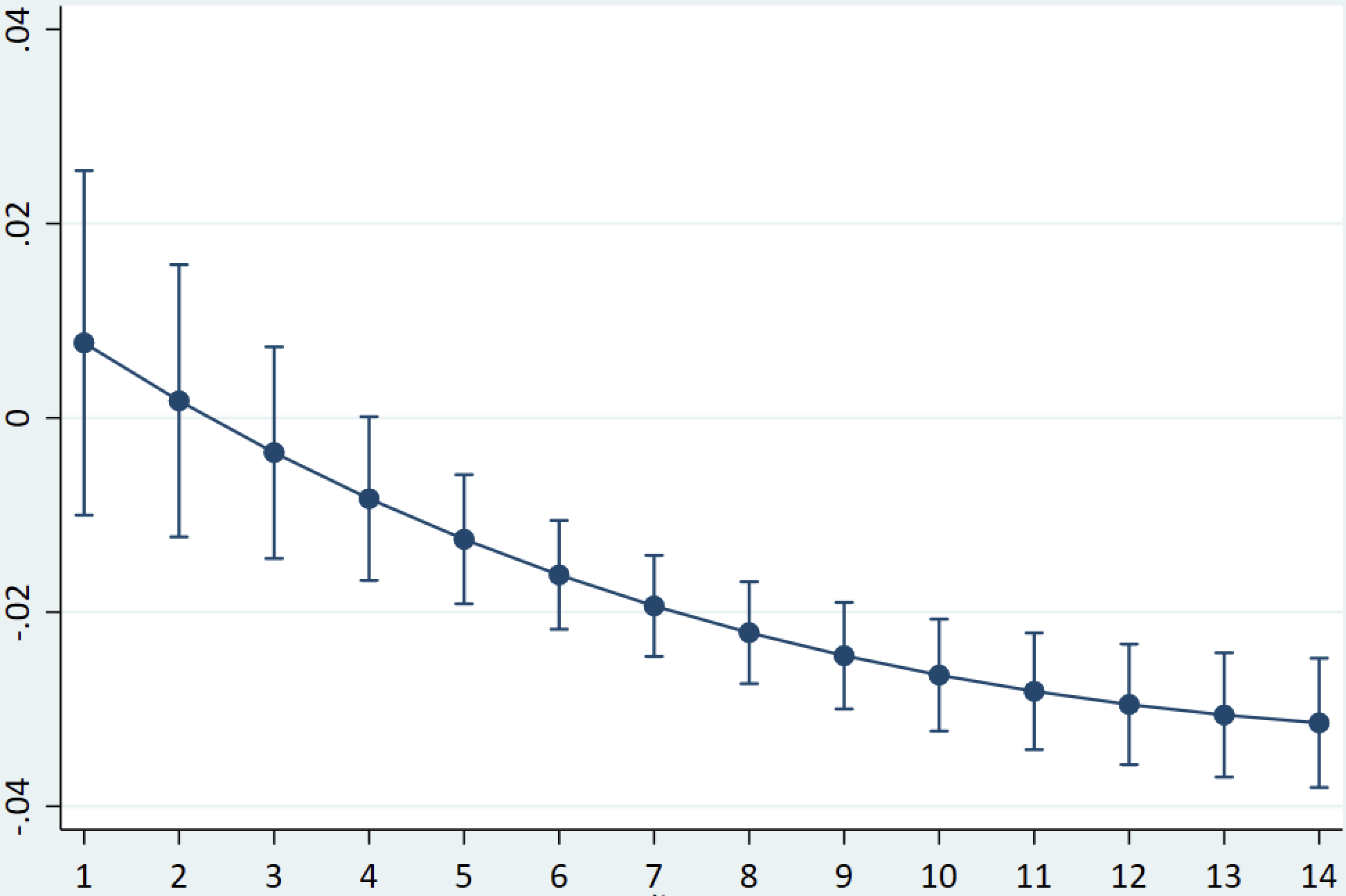 | 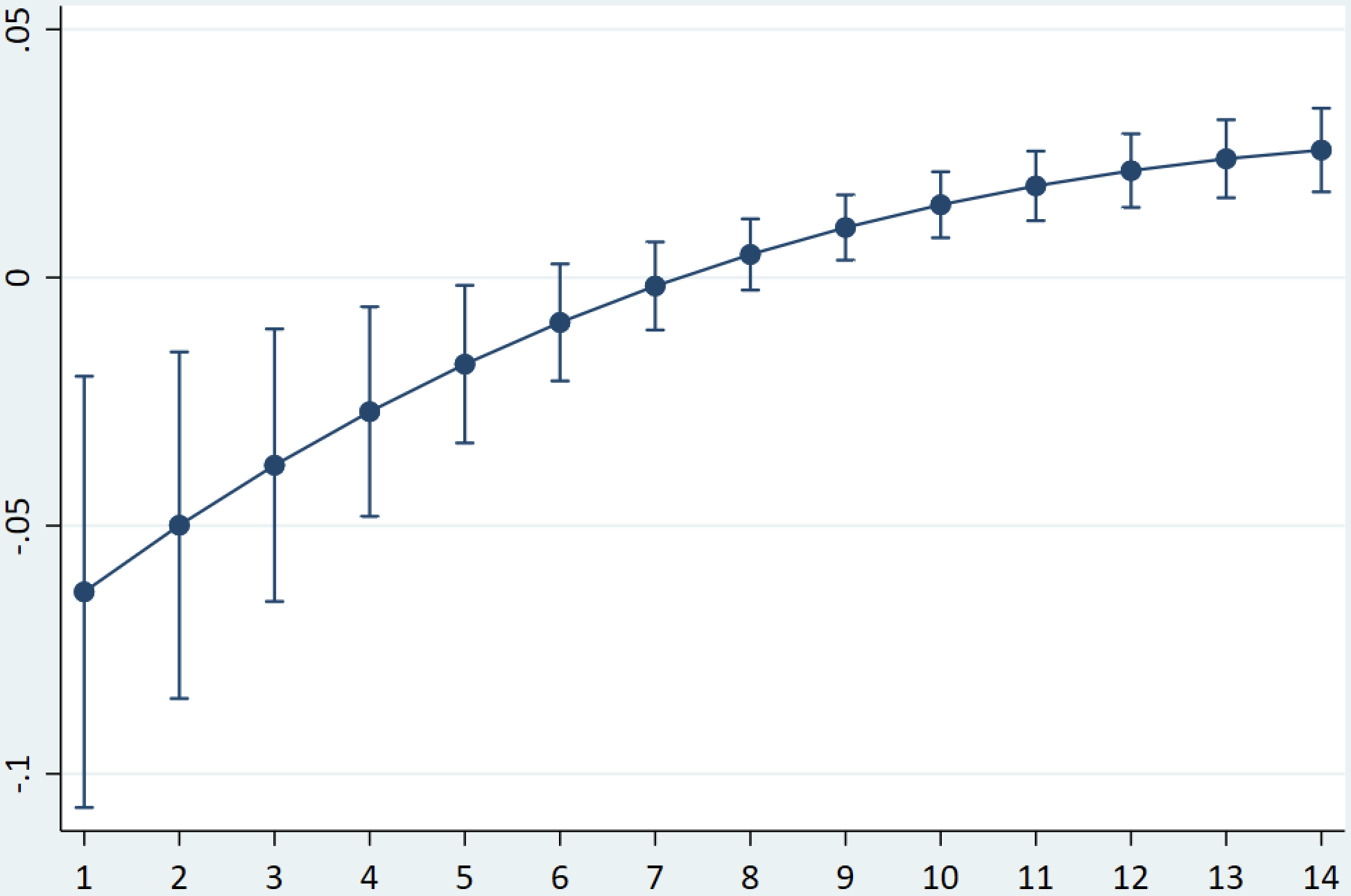 |
| Other |  |
| 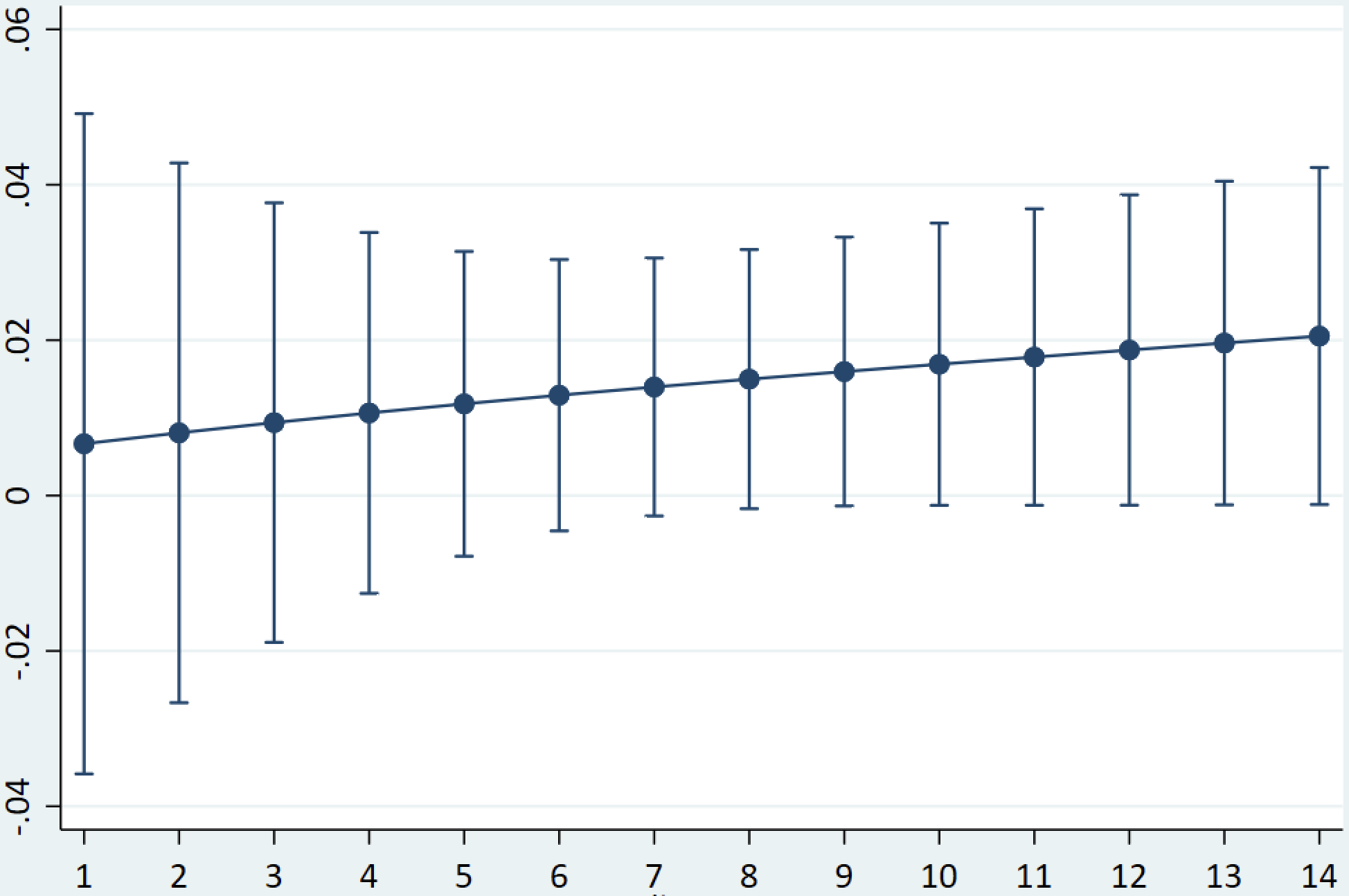 |  |

**Emotional Maturity**

| Asian | **Black** |
| --- | --- |
| 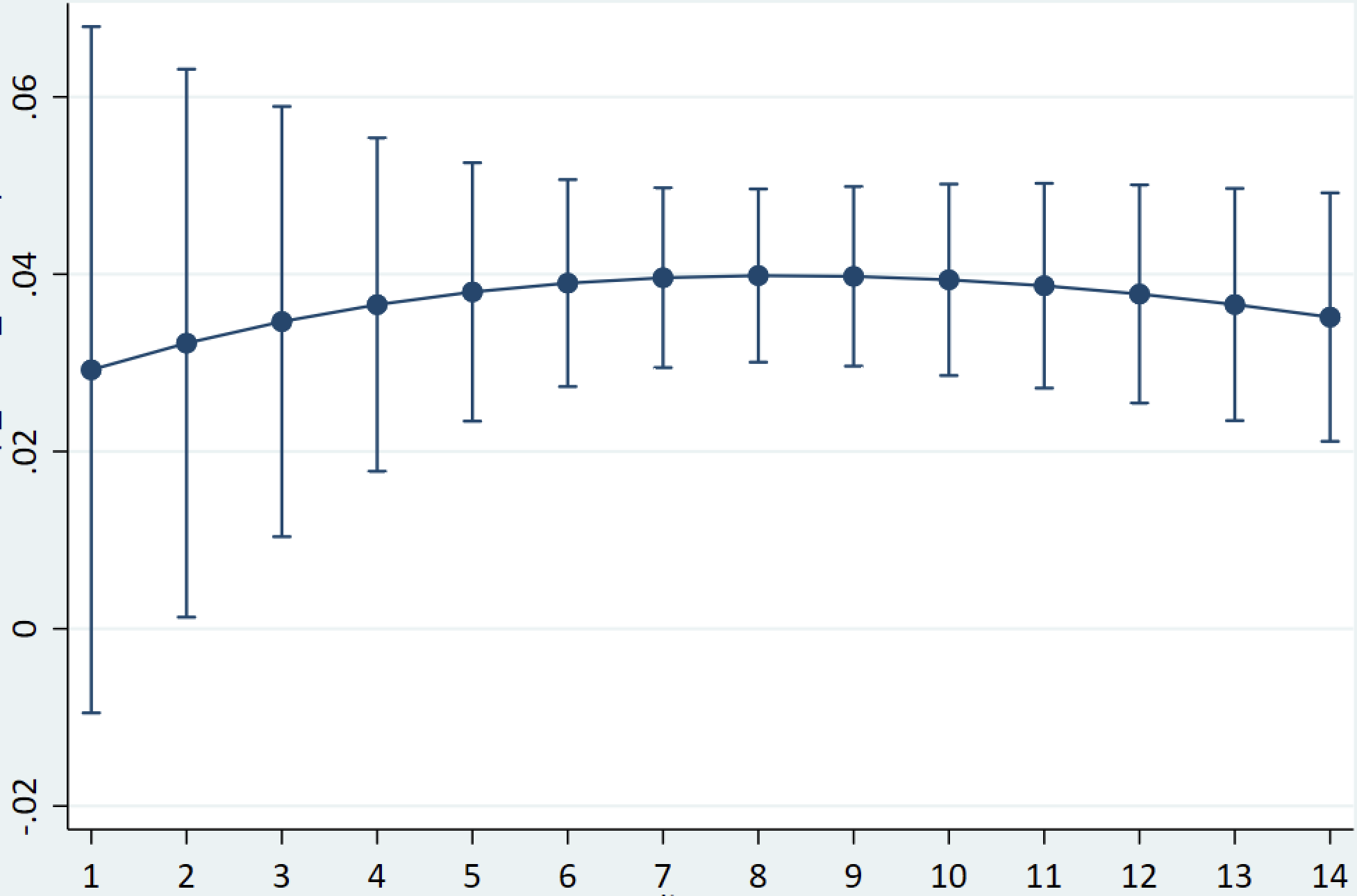 | 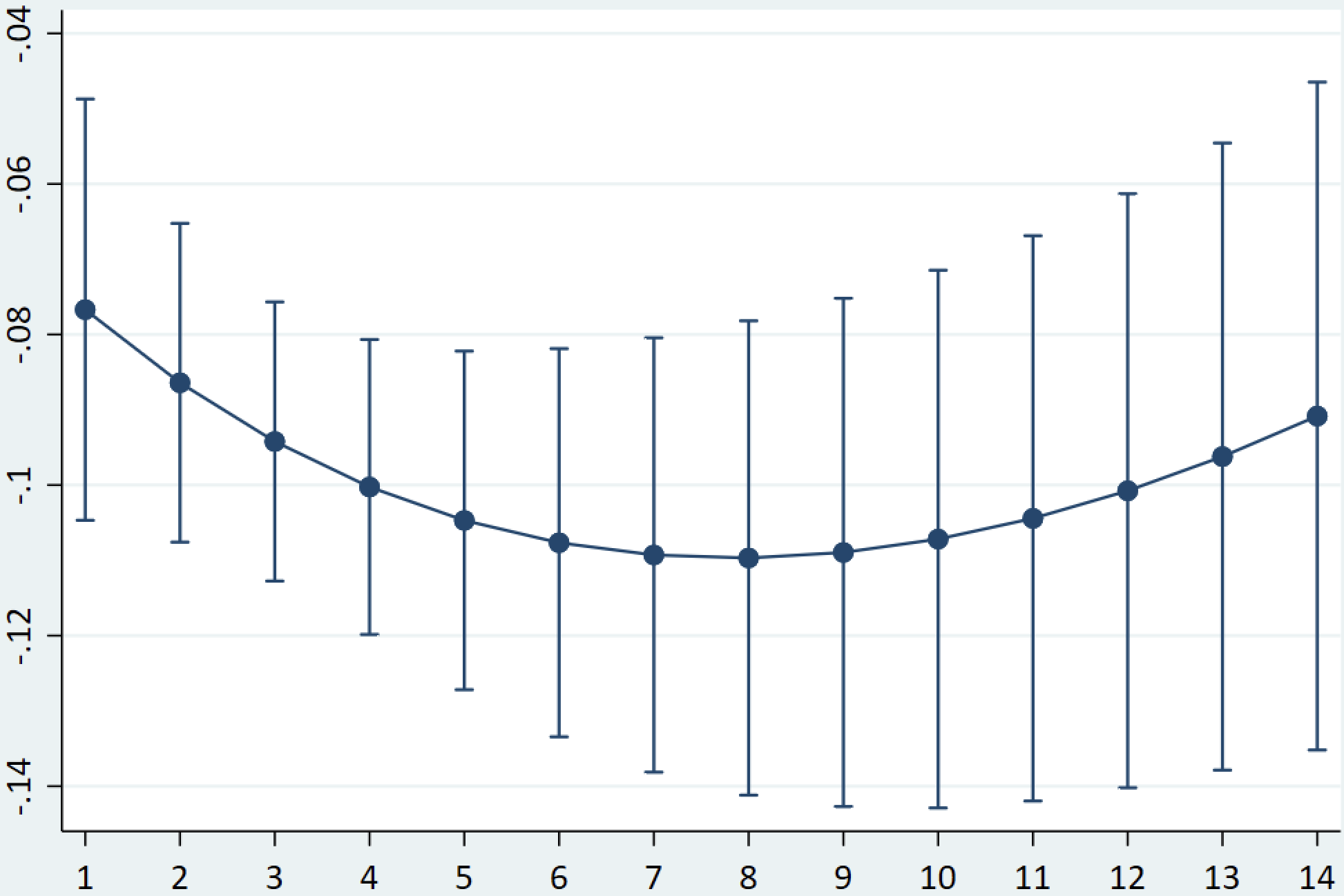 |
| Hispanic | White |
| 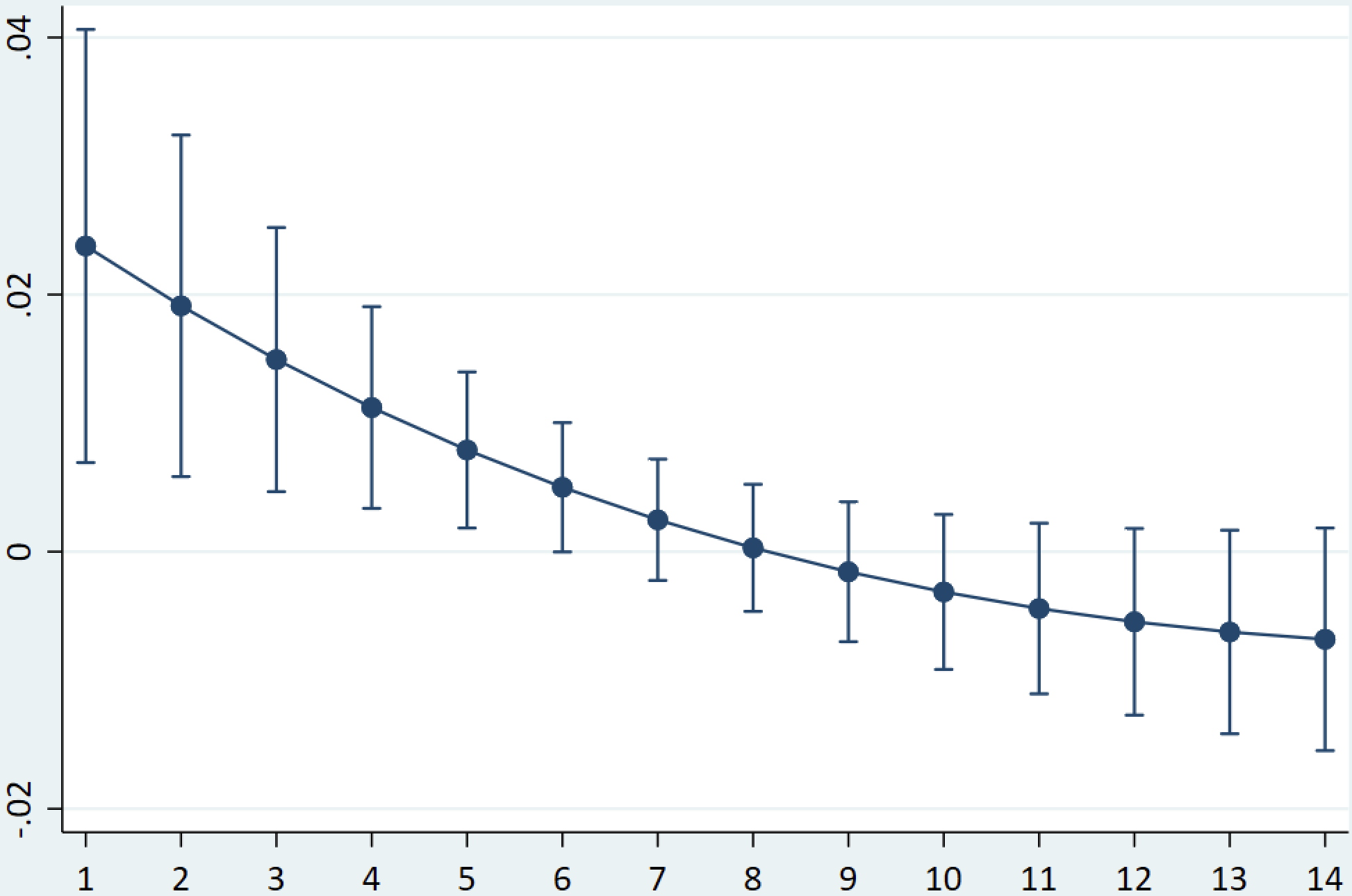 | 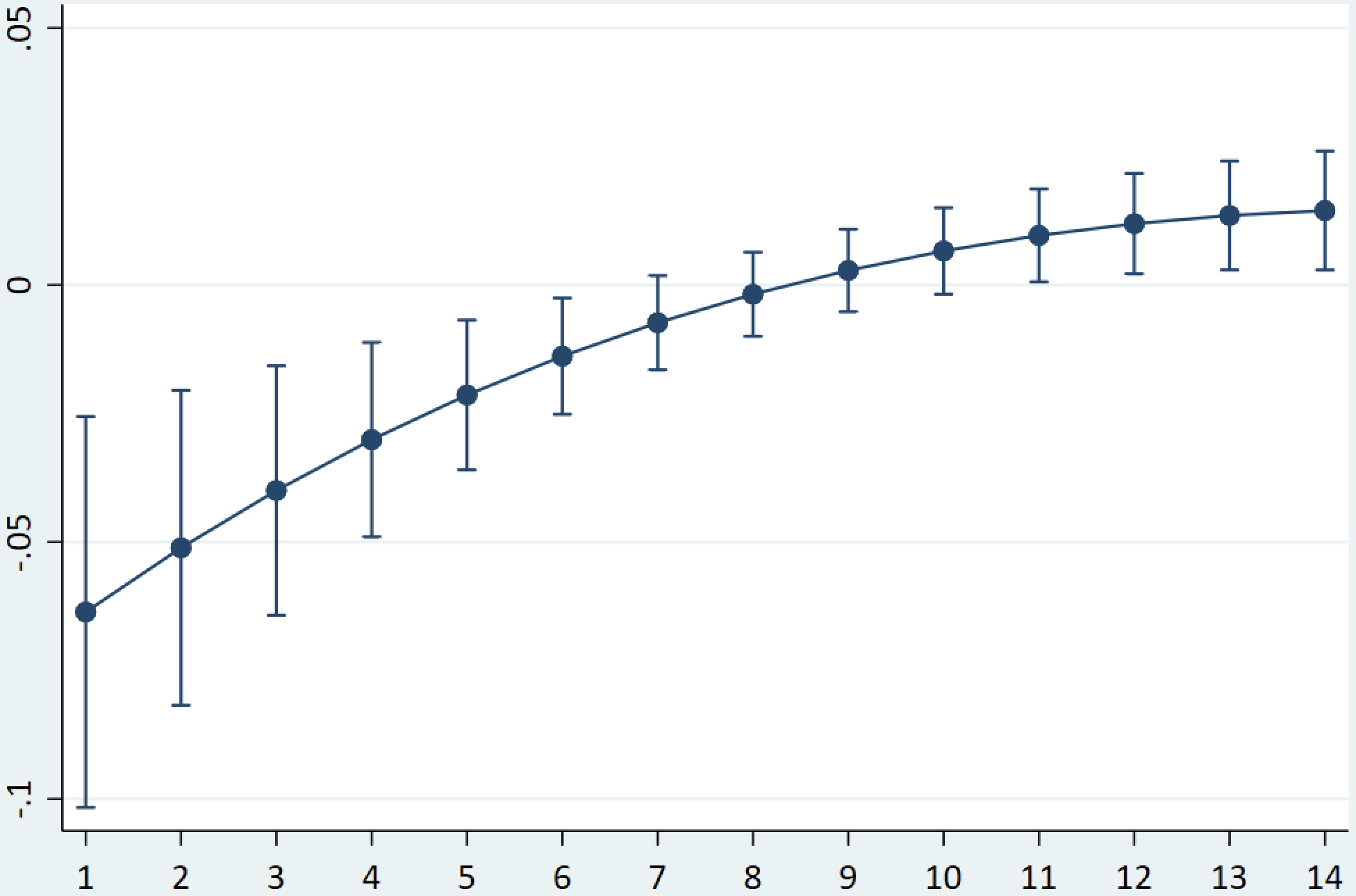 |
| Other |  |
| 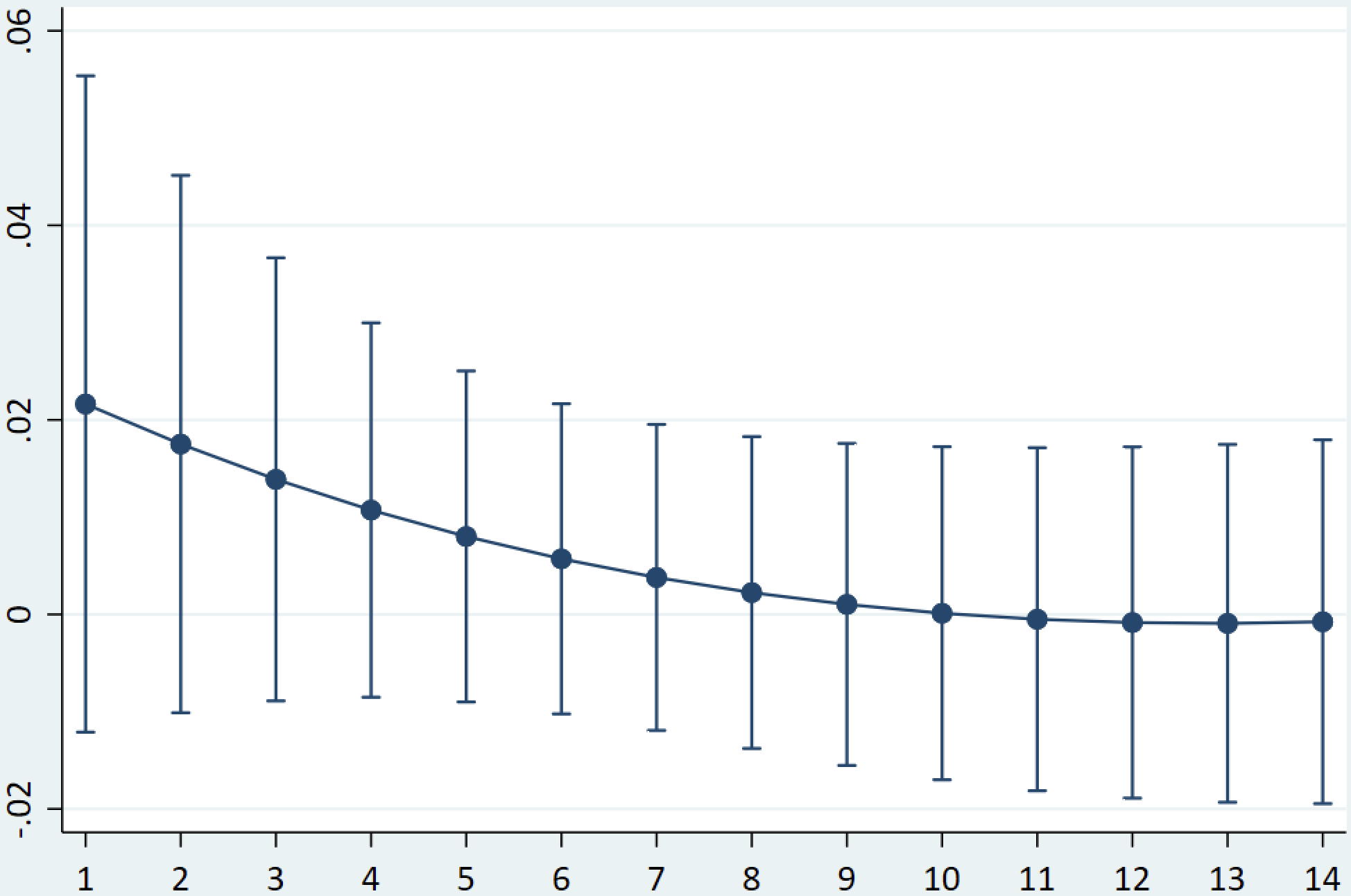 |  |

**Social Competence**

| **Asian** | **Black** |
| --- | --- |
| 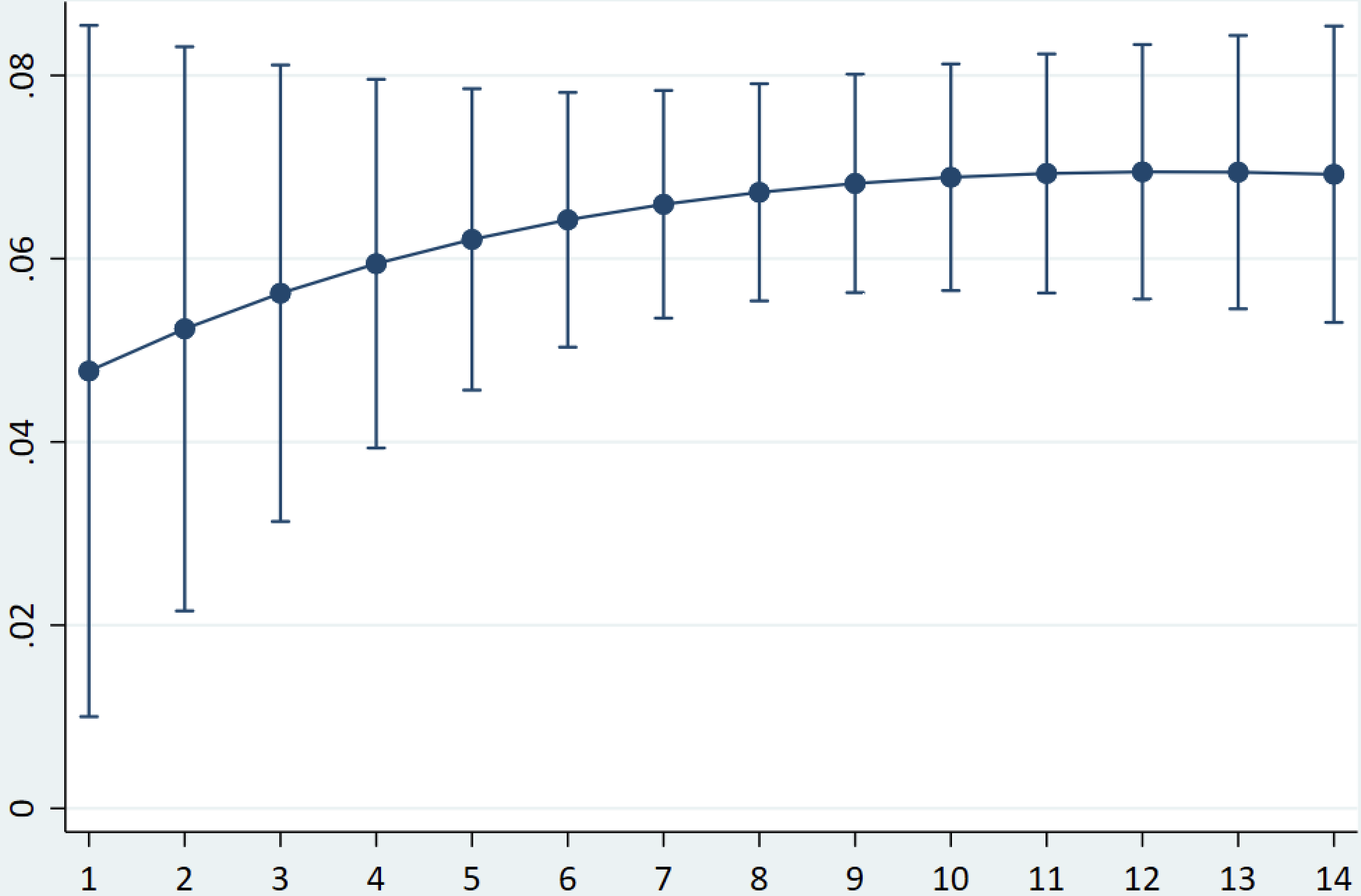 | 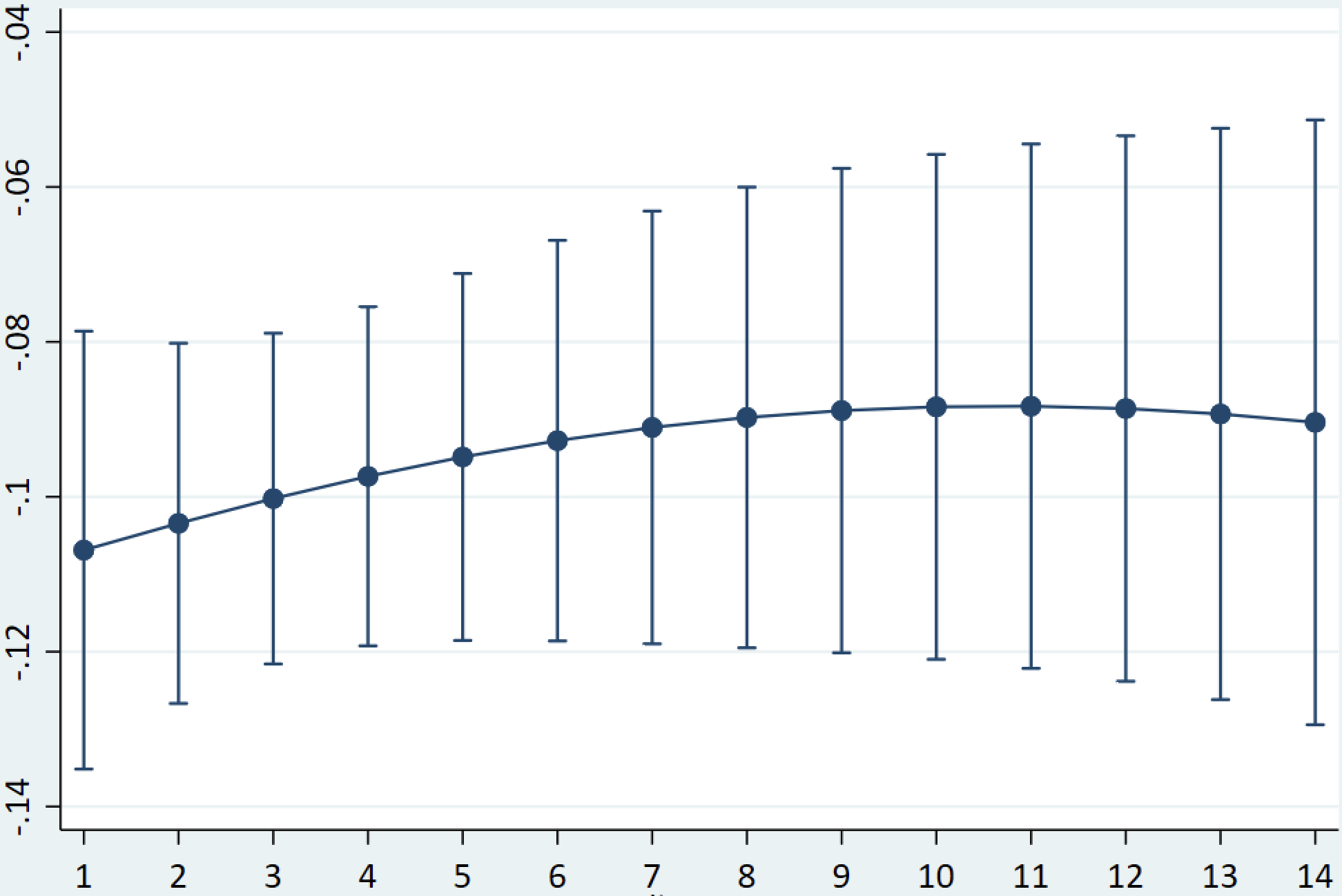 |
| Hispanic | White |
| 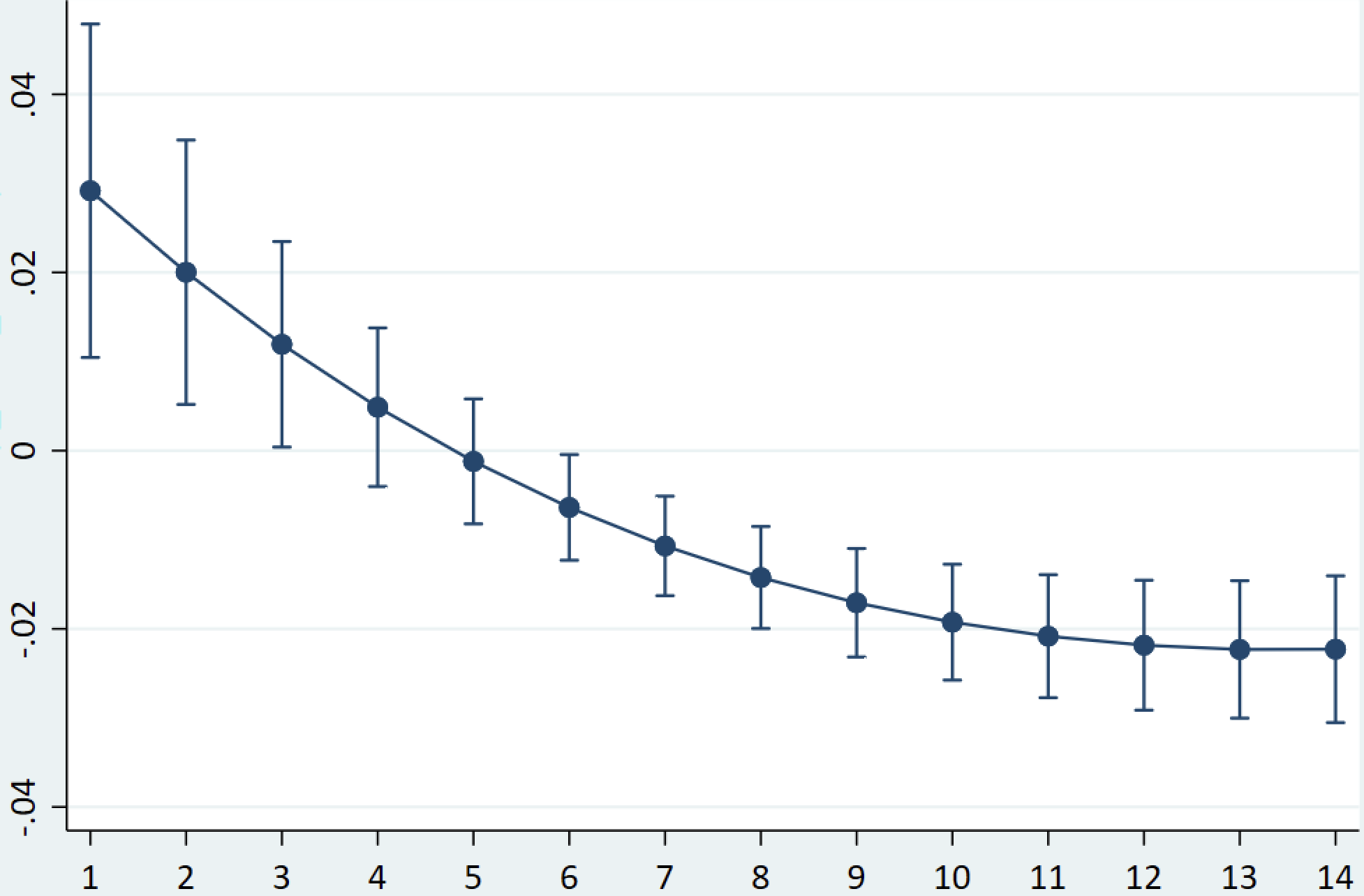 | 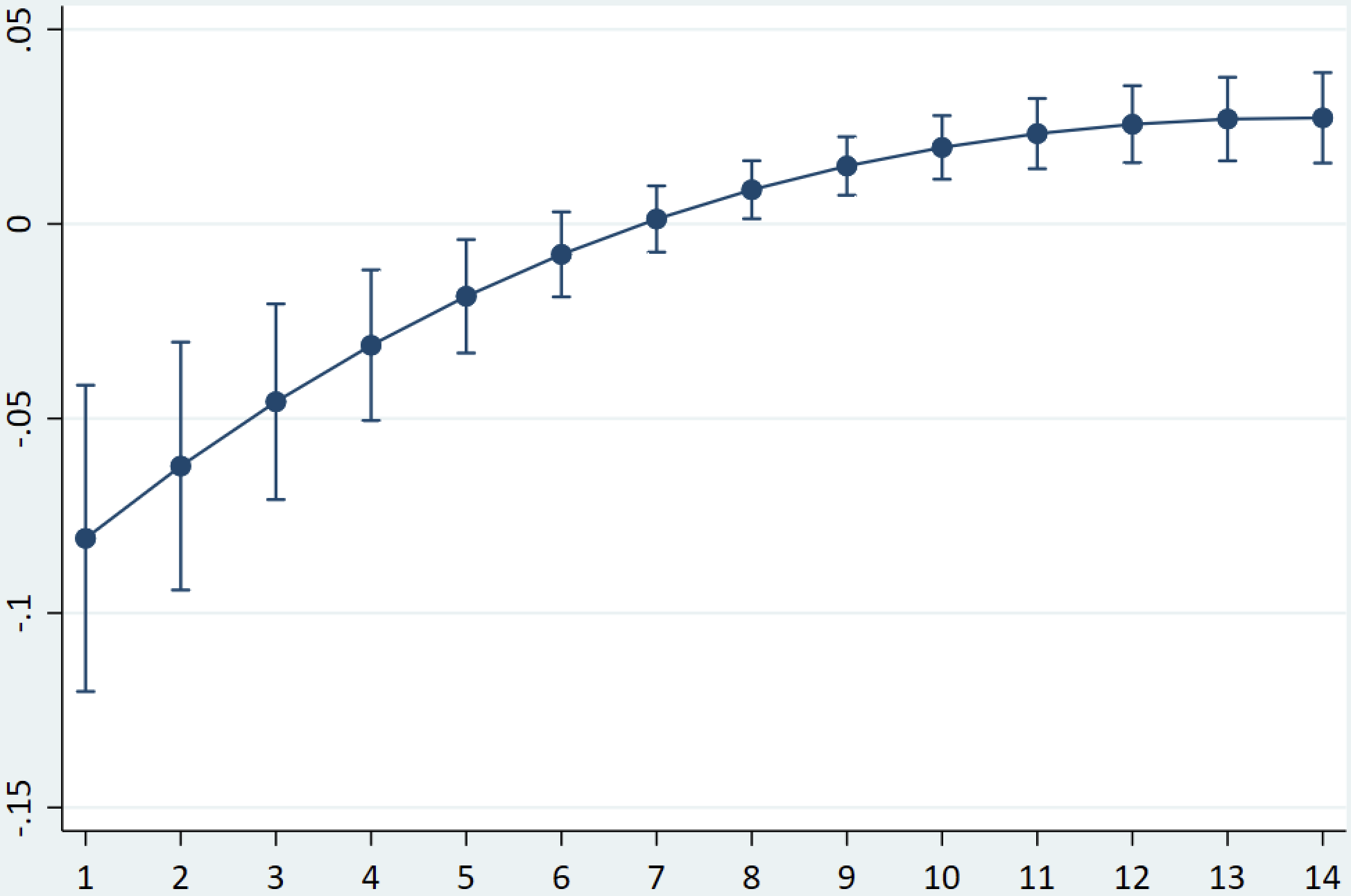 |
| Other |  |
| 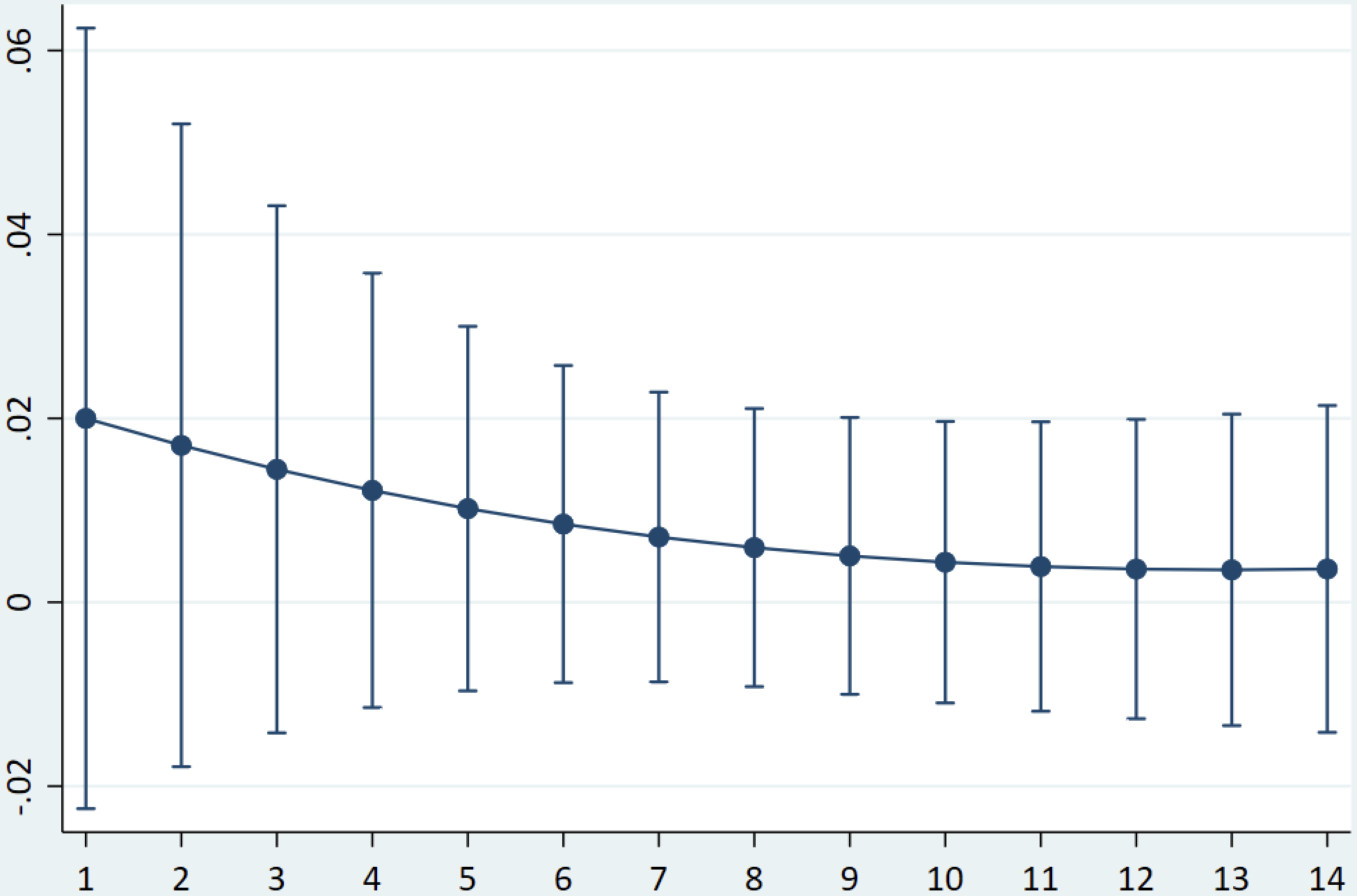 |  |

**Language and Cognition**

| **Asian** | Black |
| --- | --- |
| 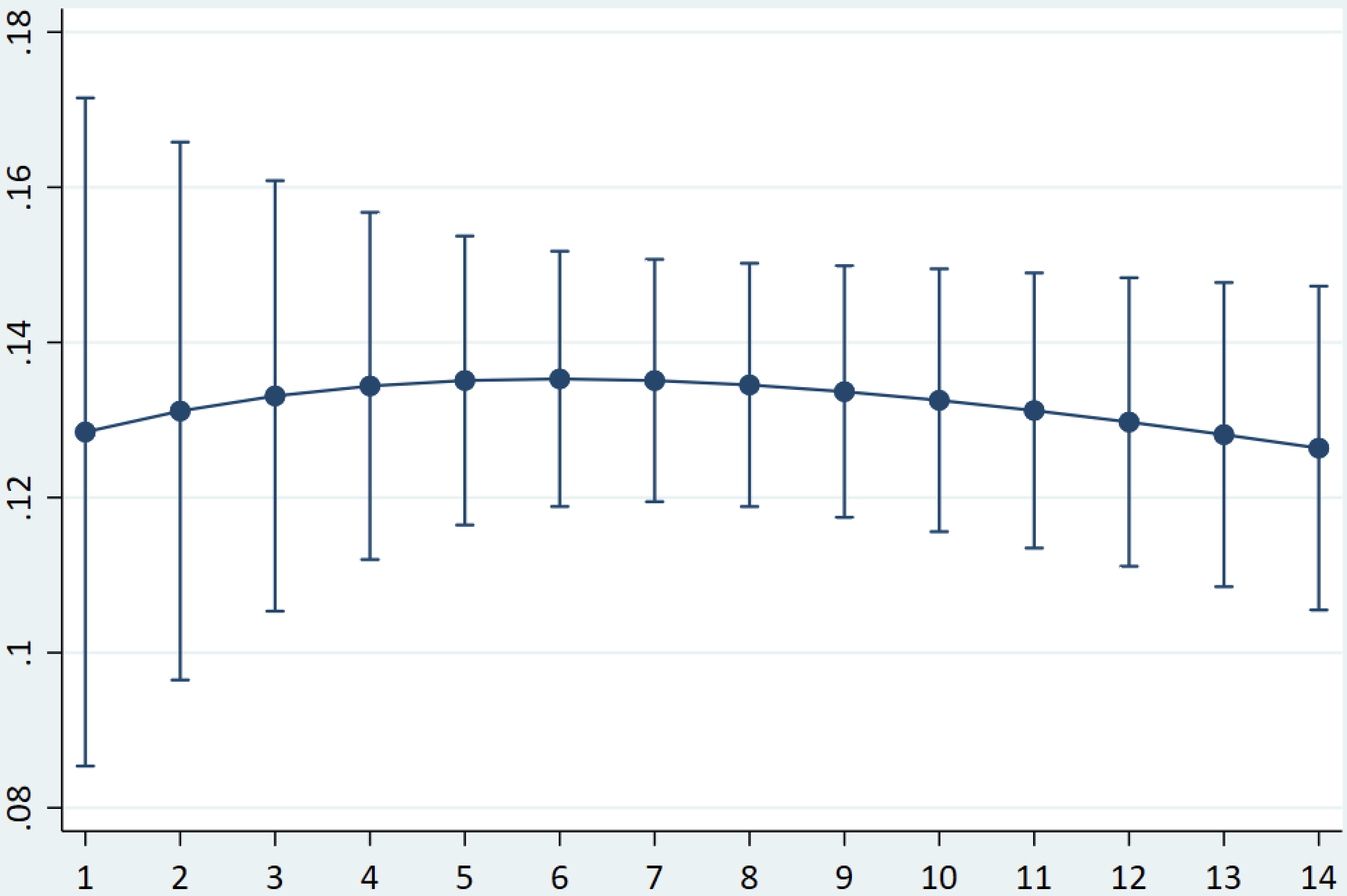 | 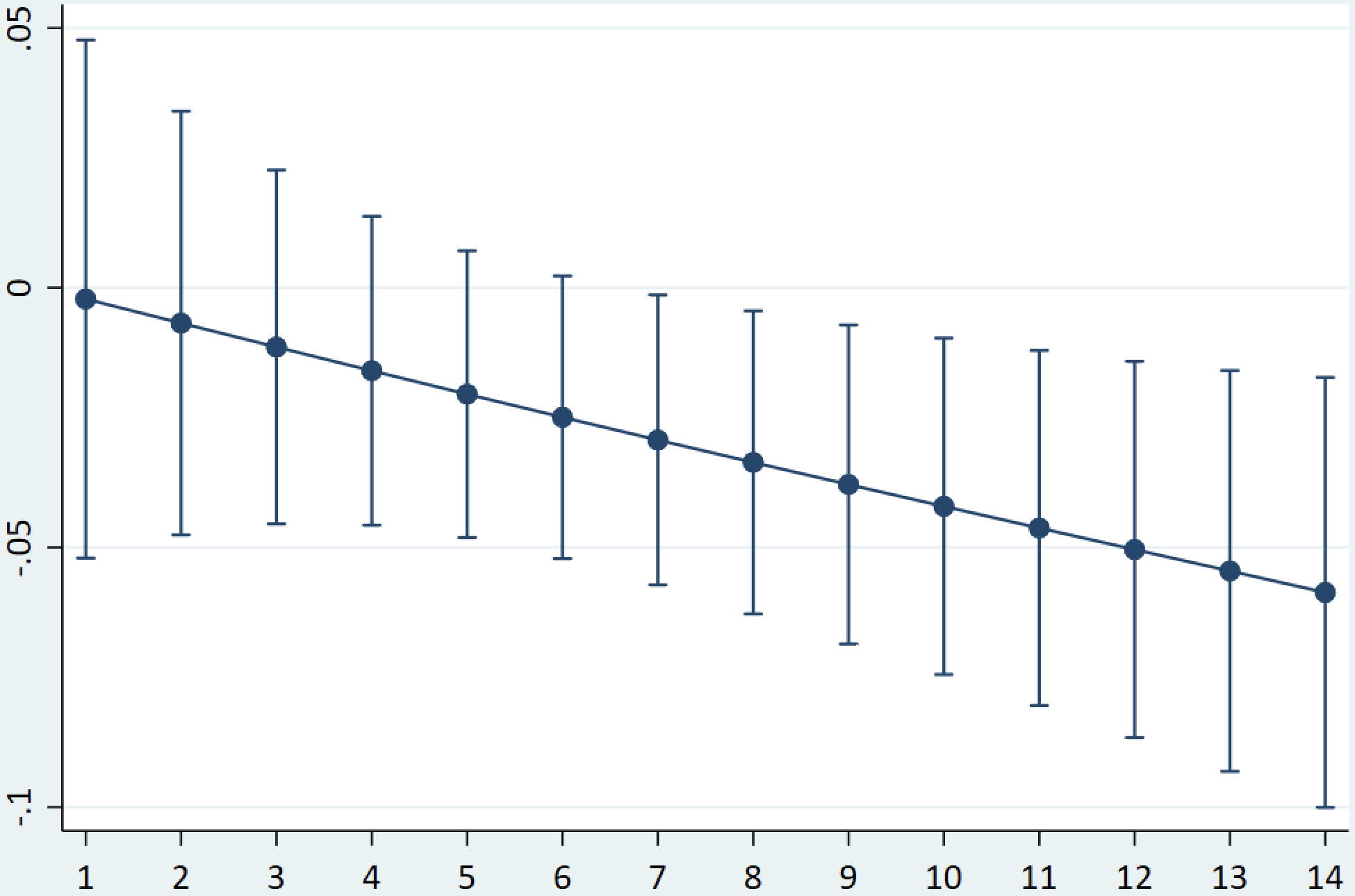 |
| **Hispanic** | White |
| 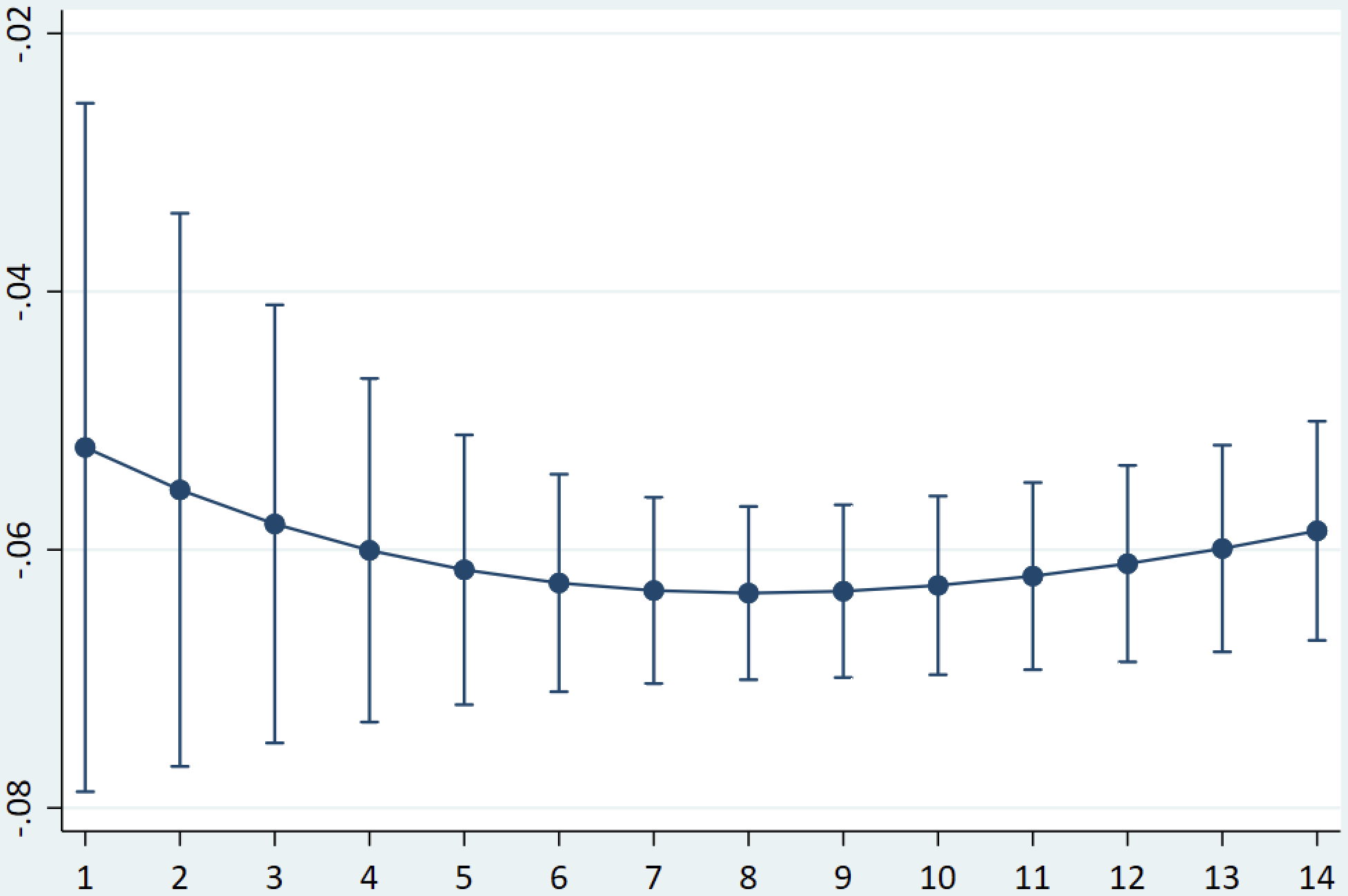 | 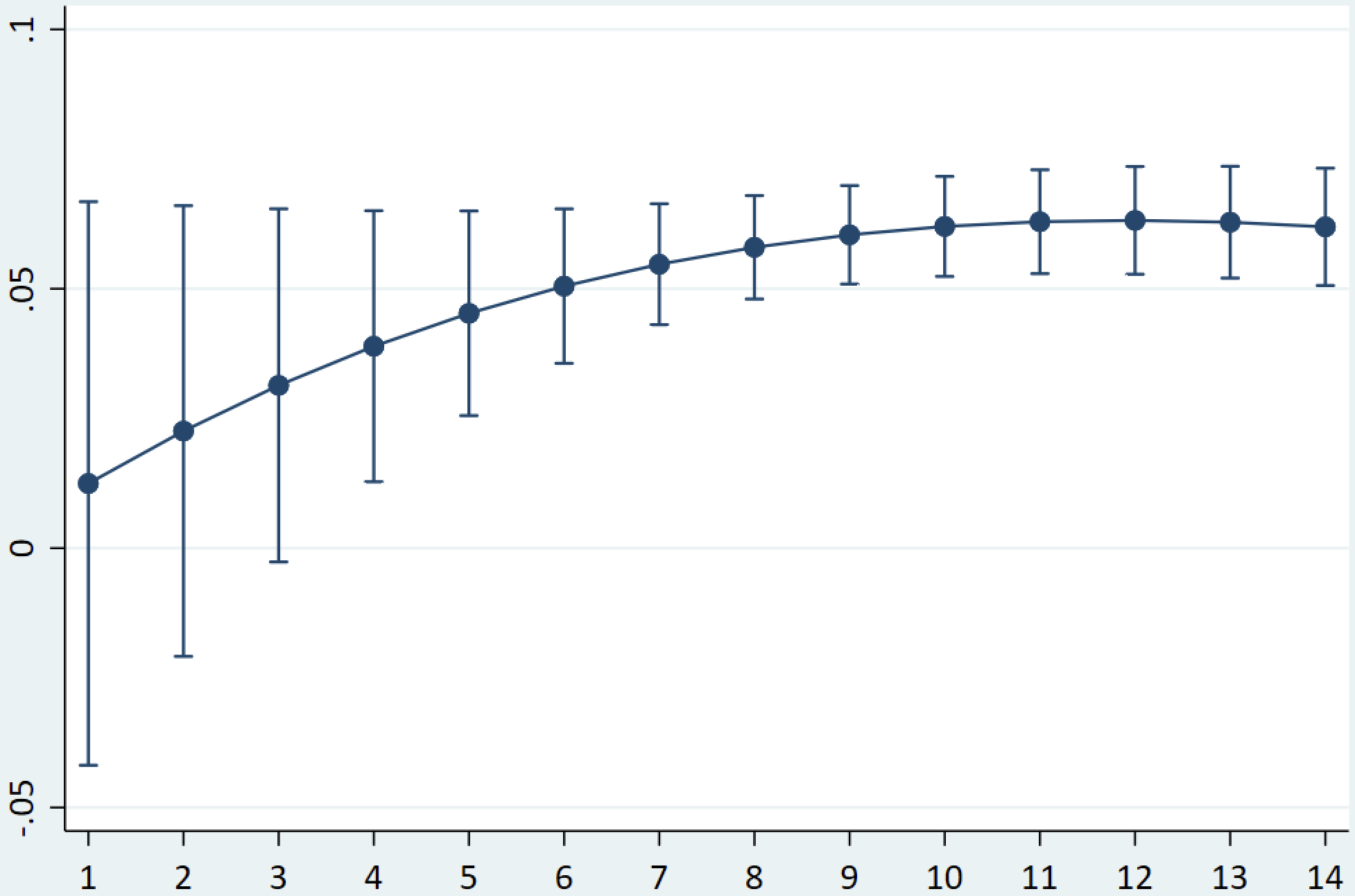 |
| Other |  |
| 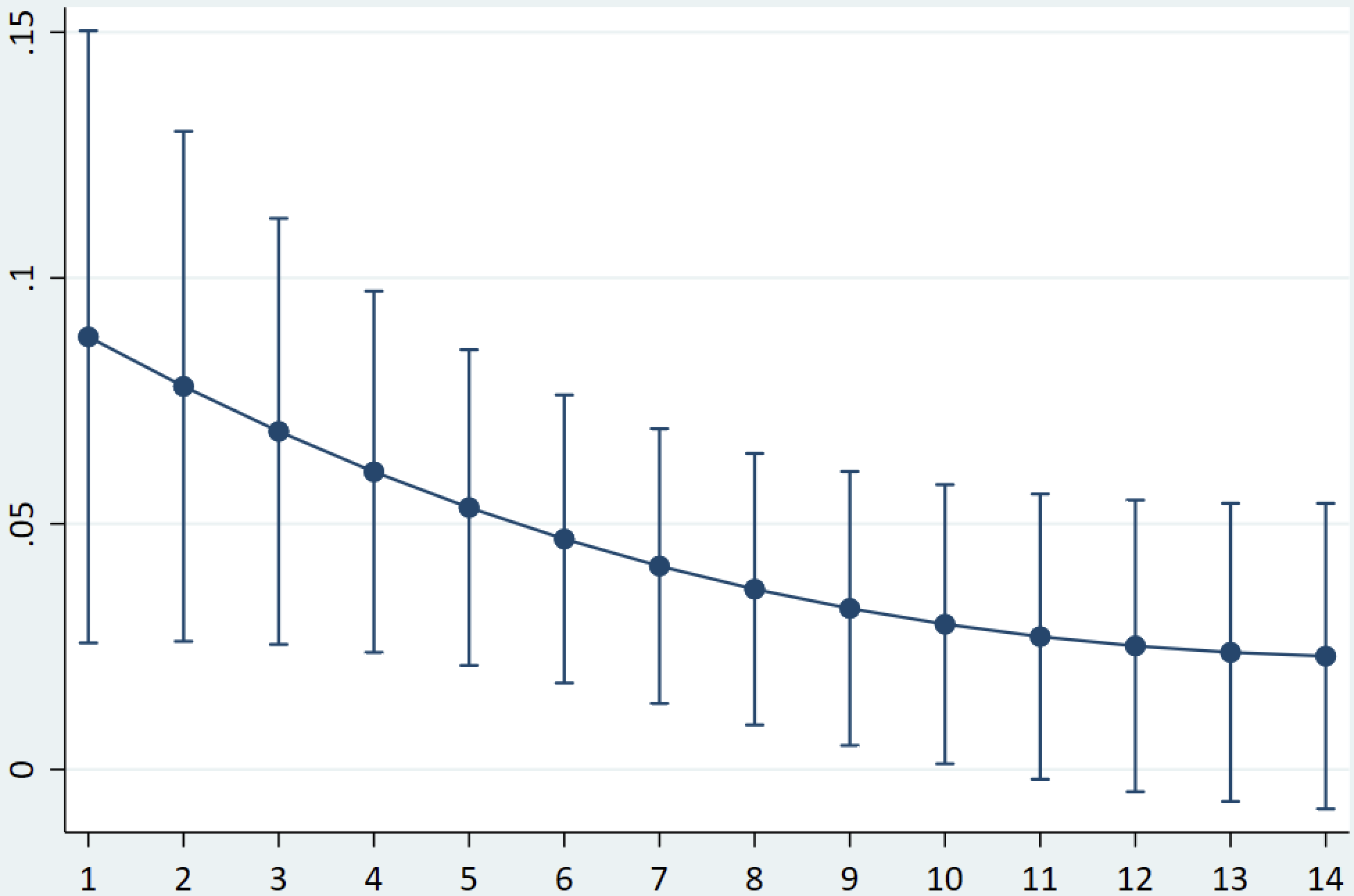 |  |

**General Knowledge**

| Asian | Black |
| --- | --- |
| 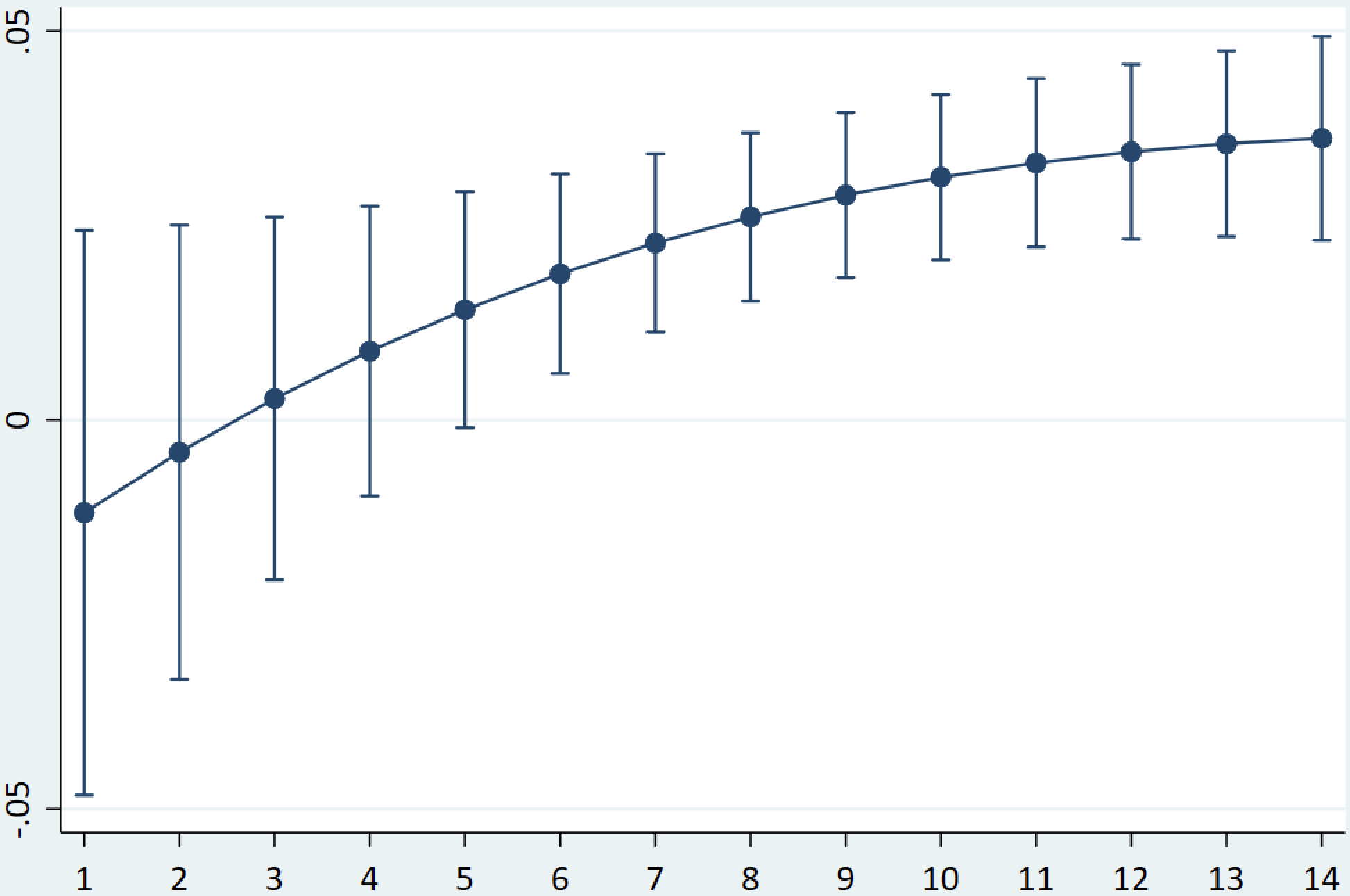 | 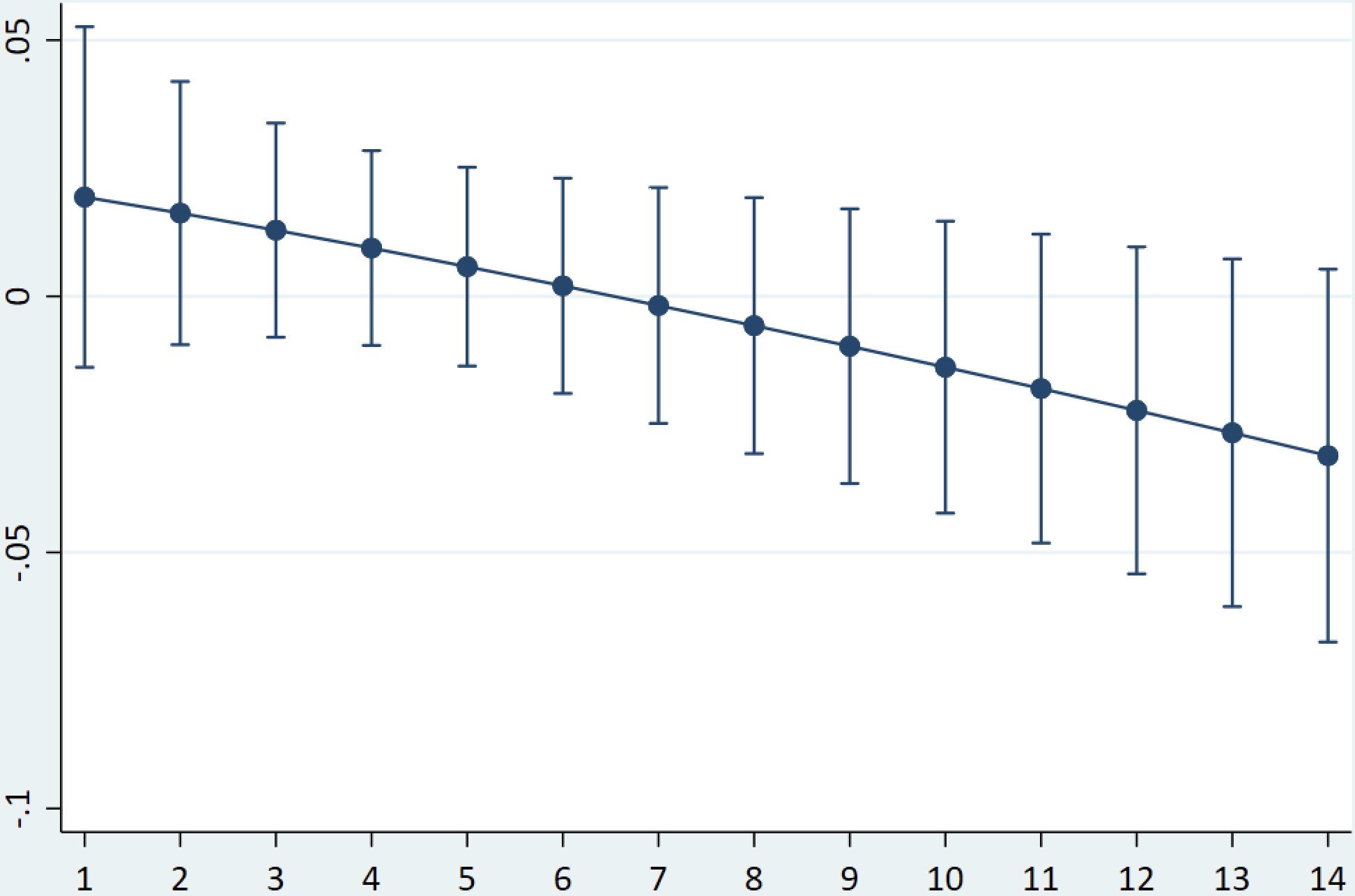 |
| **Hispanic** | White |
| 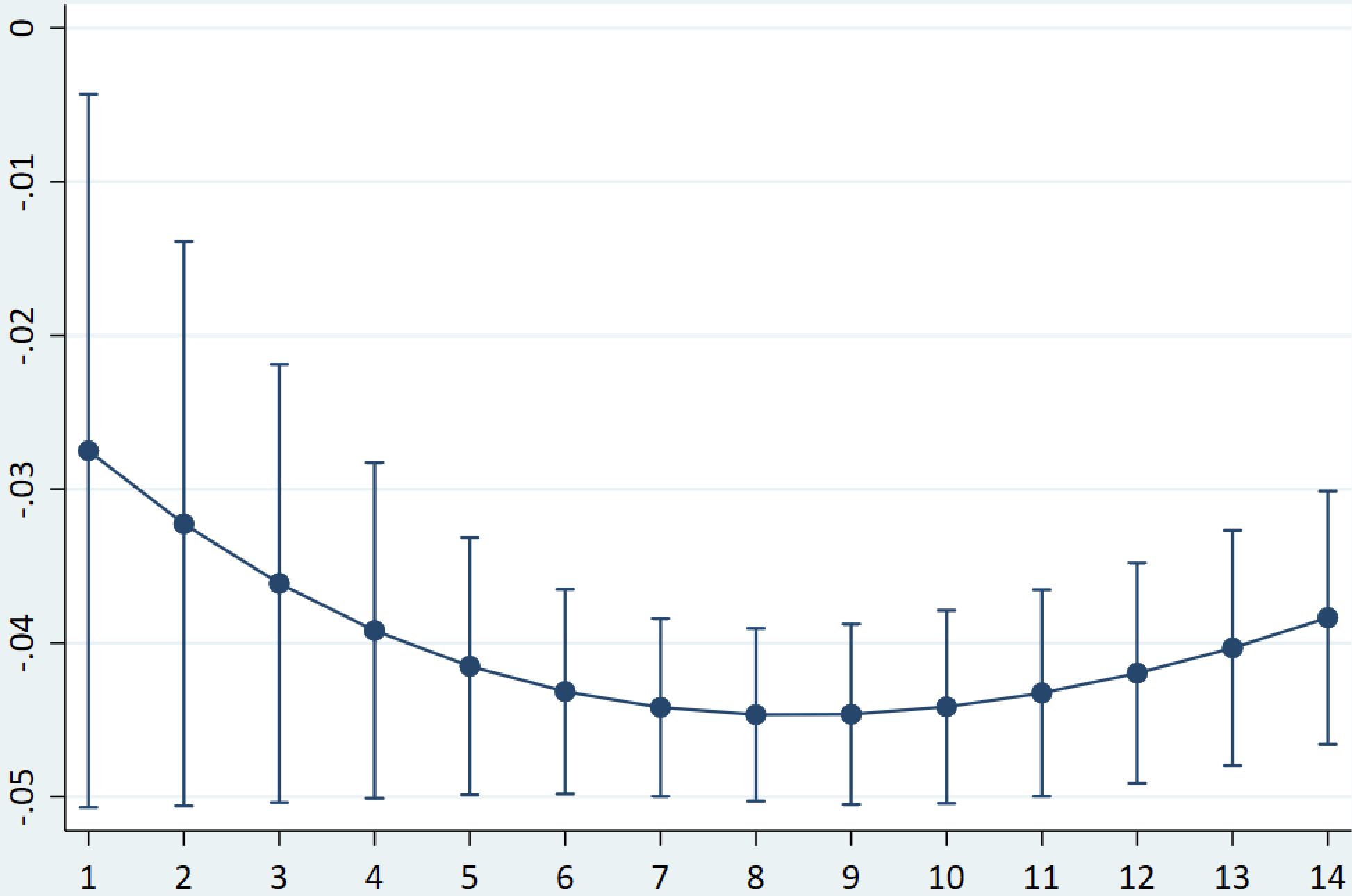 | 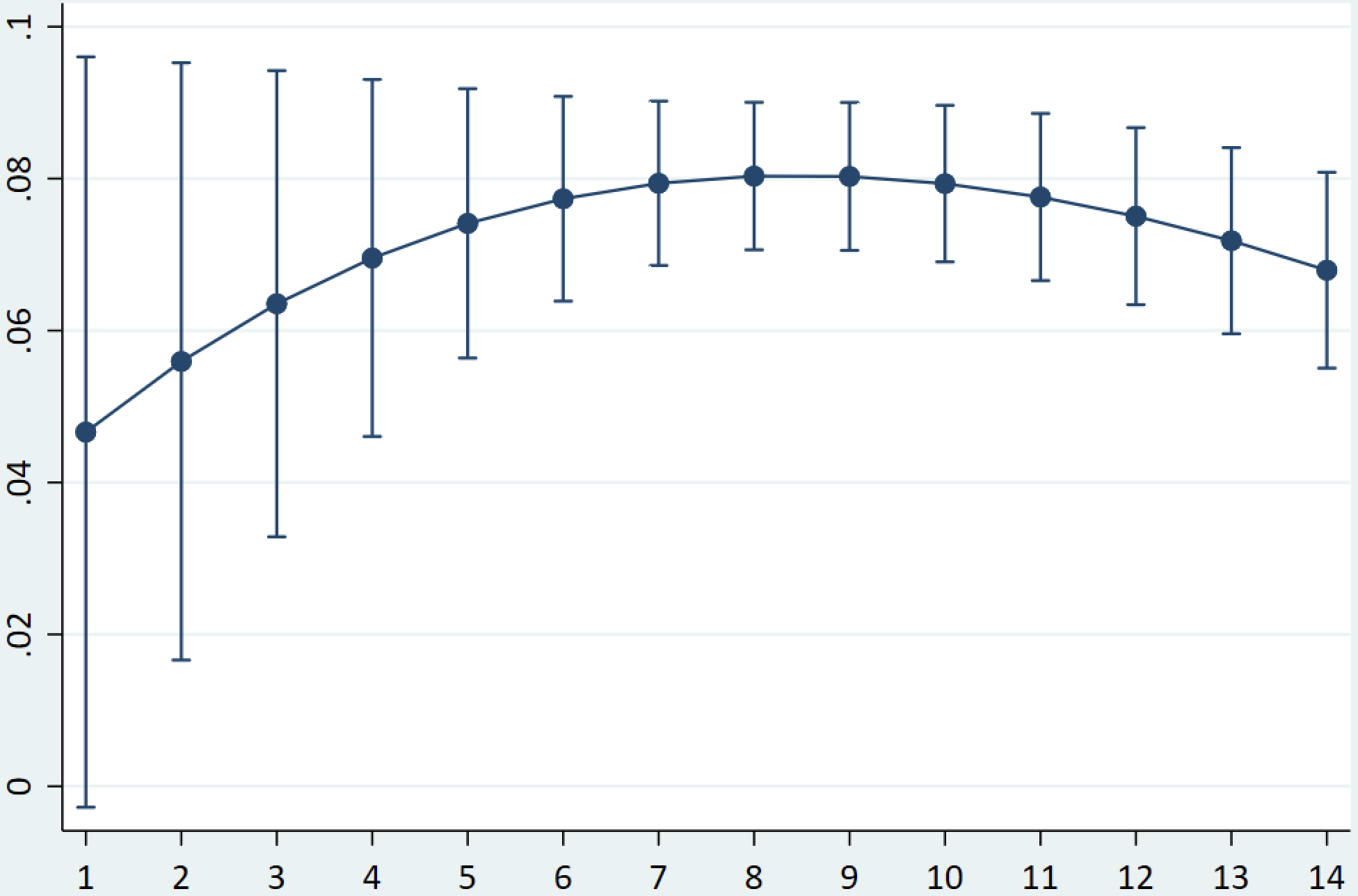 |
| Other |  |
| 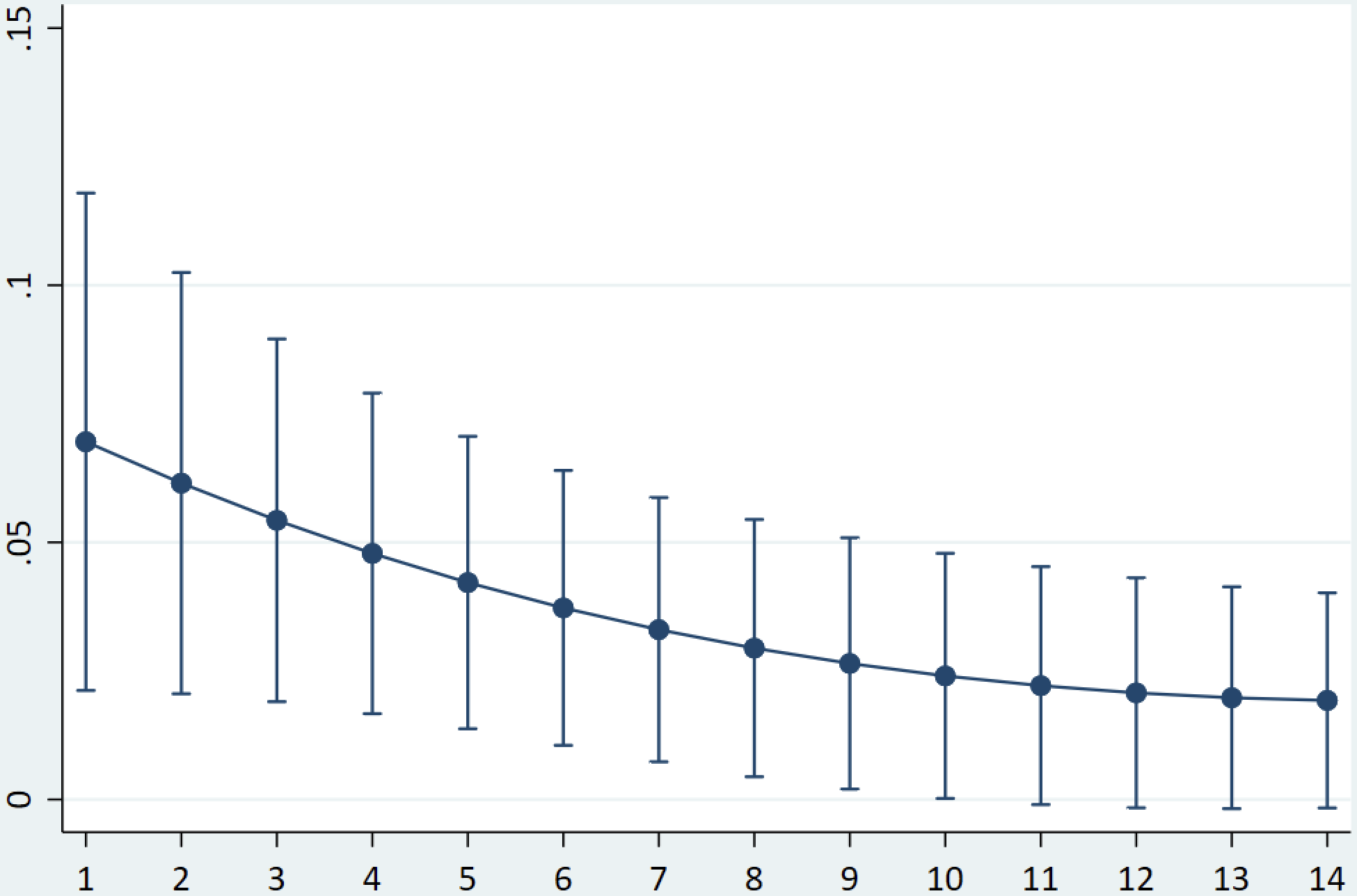 |  |
